# The DMV pore-forming TM2–Y region of SARS-CoV-2 nsp3 exhibits structural conservation beyond the coronavirus family

**DOI:** 10.1128/jvi.02038-25

**Published:** 2026-04-03

**Authors:** Alexandra Pozhidaeva, Jeffrey C. Hoch, Yulia Pustovalova

**Affiliations:** 1 Department of Molecular Biology and Biophysics, University of Connecticut Health Center 705913, Farmington, Connecticut, USA; Loyola University Chicago - Health Sciences Campus, Maywood, Illinois, USA

**Keywords:** SARS-CoV-2, nsp3, Y domain, double-membrane vesicle, DMV pore, MD simulation, AlphaFold

## Abstract

Positive-strand RNA viruses remodel host intracellular membranes to form replication organelles in the cytoplasm. SARS-CoV-2 and other coronaviruses, members of the *Nidovirales* order, replicate within double-membrane vesicles (DMVs) derived from the endoplasmic reticulum that protect newly synthesized viral RNA (vRNA). A recently discovered SARS-CoV-2 molecular pore complex composed of non-structural proteins 3 and 4 (nsp3 and nsp4) connects the DMV interior with the cytoplasm, enabling vRNA export for subsequent packaging into virions. The C-terminal region of nsp3, the Y domain, oligomerizes to form the base of the cytosolic crown of this pore, while the preceding transmembrane domain 2 (TM2) anchors the pore in the DMV membrane. However, the molecular mechanisms underlying pore formation and whether similar structures are conserved across other *Nidovirales* members remain unclear. Here, we combined AlphaFold structure prediction, structure-informed multiple sequence alignments, and molecular dynamics simulations to investigate the conservation and potential function of the C-terminal TM2-Y region of nsp3 across nidoviruses. We show that despite minimal sequence identity, all modeled TM2-Y regions adopt similar domain folds. In particular, the wedge-shaped TM2, the Zn-binding Y1 subdomain, and its adjacent Y2 subdomain constitute a conserved structural module present in most vertebrate-infecting *Nidovirales* families, including *Coronaviridae*, *Tobaniviridae*, *Gresnaviridae*, *Olifoviridae,* and *Arteriviridae*. Across these viruses, Zn(II) is coordinated with a distinctive octahedral geometry involving two water molecules. This conserved architecture likely represents an evolutionarily maintained mechanism for DMV pore formation, in which the Zn-binding region engages the membrane surface to induce local lipid bilayer destabilization that may precede DMV pore assembly.

**IMPORTANCE:**

Over the last 25 years, several coronaviruses, including highly pathogenic SARS-CoV, MERS-CoV, and SARS-CoV-2, have emerged in humans through zoonotic transmission. With nearly 100 coronaviruses known, further spillovers remain possible. Coronaviruses and other nidoviruses replicate within specialized replication organelles formed from host membranes; however, how they form remains poorly understood. This study reveals that coronaviruses and distantly related tobaniviruses and arteriviruses might share a conserved molecular framework for assembling membrane pores that connect the DMV interior, where vRNA is synthetized, and the cytoplasm, where virions are assembled. By identifying conserved structural elements in the C-terminal region of nsp3, including wedge-shaped TM2 domain and Zn-binding Y1/Y2 tandem, we show that vertebrate-infecting nidoviruses likely share structural mechanism for DMV pore formation. These findings provide insight into how diverse nidoviruses remodel host membranes and highlight the C-terminal region of nsp3 as a potential target for broad-spectrum antivirals.

## INTRODUCTION

**Fig 1:**
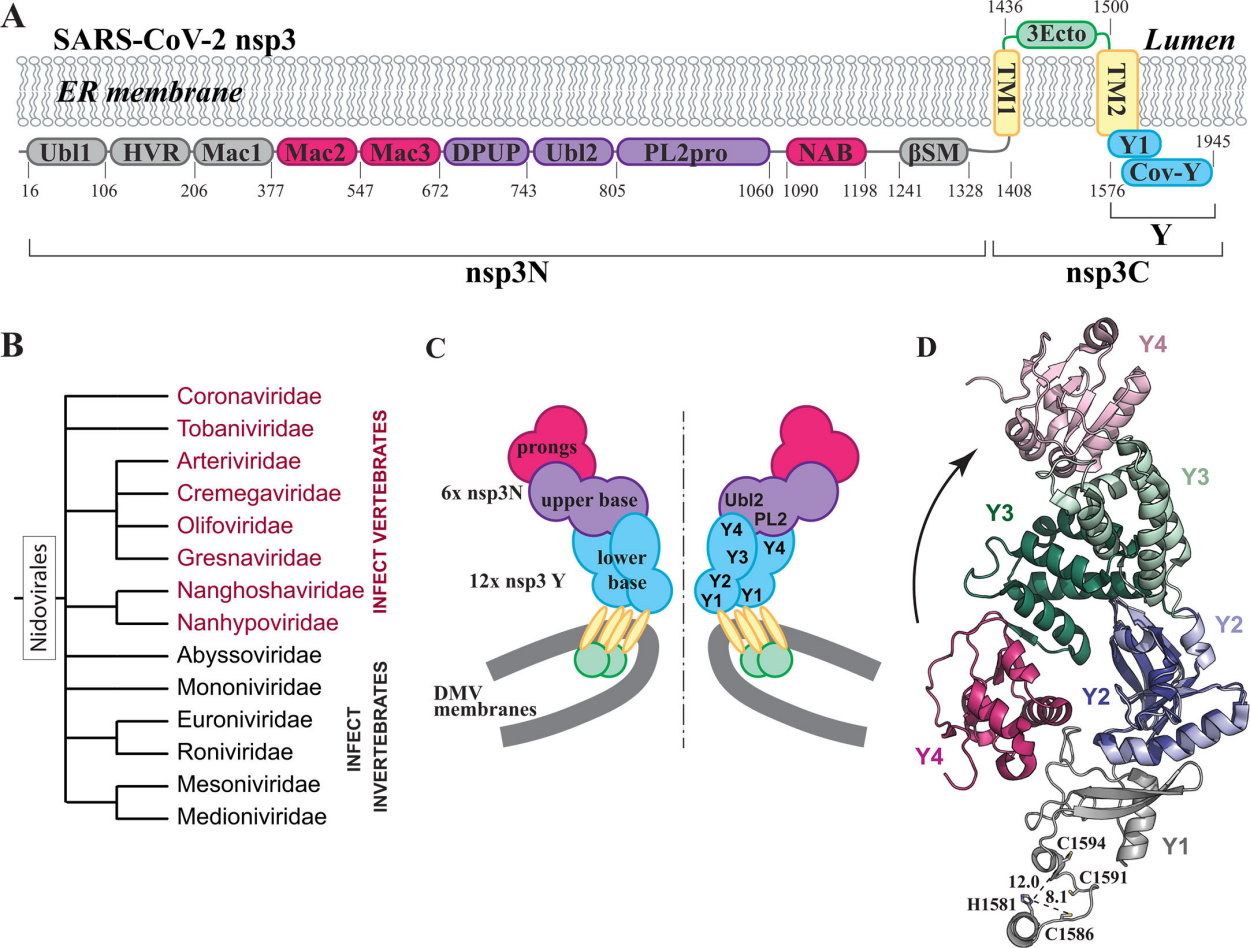
Overview of nsp3 domain organization and DMV molecular pore architecture. (**A**) Domain organization of SARS-CoV-2 nsp3, with individual domains colored according to their spatial positions within the DMV pore crown shown in panel C. Domains colored gray were not resolved in the cryo-EM structure of the pore by Huang et al. (9). (**B**) Current classification of the order *Nidovirales*. The order comprises 8 suborders and 14 families. (**C**) Architecture of the DMV molecular pore crown formed by nsp3, adapted from Huang et al. (9). (**D**) Structural overlay of SARS-CoV-2 nsp3 CoV-Y from the crystal structure (PDB: 8F2E, shown in darker colors) and from the cryo-EM structure of the DMV pore (PDB: 8YAX, shown in lighter colors). Side chains of Zn-BM1 coordinating residues (H1581, C1586, C1591, and C1594) are shown as sticks. Dashed lines indicate distances between Zn-coordinating atoms, labeled in Å, illustrating the loss of proper Zn(II) coordination geometry in the assembled pore.

Highly pathogenic coronaviruses (CoVs) such as severe acute respiratory syndrome (SARS-CoV) (1, 2), Middle East respiratory syndrome-related coronavirus (MERS-CoV) (3), and the recently emerged SARS-CoV-2 that causes COVID-19 (4–6) have resulted in millions of fatalities worldwide. Together with four other coronaviruses known to cause “common cold” in humans (HCoV-229E, HCoV-NL63, HCoV-HKU1, and HCoV-OC43) (7), these viruses are classified under the *Orthocoronavirinae* subfamily, within the *Coronaviridae* family of order *Nidovirales* (8). The order includes thirteen additional virus families that infect vertebrate and invertebrate hosts, collectively representing one of the most diverse groups of RNA viruses (Fig. 1B). Despite their diversity, all nidoviruses share a similar genomic organization and replication strategy (8).

All nidoviruses are positive-sense single-stranded RNA (+ssRNA) viruses, with most sharing a genome organization composed of multiple open reading frames (ORFs). Non-structural proteins (nsps), which are essential for genome expression and replication, are encoded in two overlapping ORFs, ORF1a and ORF1b, situated at the 5′-terminal two-thirds of the viral genome. The nsps are transcribed as polyproteins, pp1a and pp1ab, and subsequently derived via autoproteolysis. Coronaviruses produce 15 or 16 nsps (nsp1-15/16), while toroviruses from the *Tobaniviridae* family generate 13, and arteriviruses produce 12 (10, 11). The exact number of nsps in other less characterized nidoviruses remains undetermined; however, five nsps with seven distinct domains are essential for genome replication and are conserved across all nidoviruses (10).

Nsps assemble into a replication-translation complex (RTC) responsible for vRNA synthesis. The common feature of +ssRNA viruses is that RTCs are assembled inside membrane-bound organelles, which protect viral replication from host immune defenses (12). Morphology of these replication compartments and host cell membranes that are repurposed for their construction can vary (13, 14). Coronaviruses, along with toroviruses and arteriviruses, hijack the host endoplasmic reticulum (ER) membrane to build an interconnected network of double membrane vesicles (DMVs) (15–17). DMV formation is a complex process involving substantial host membrane rearrangement and changes in lipid composition, and many aspects of this process remain poorly understood.

Following synthesis, vRNA must be exported from the DMV to the cytoplasm for packaging into viral particles. However, until recently, it was unclear if the DMV interior was connected to the cytosol. In a pioneering observation, Wolff et al. identified a molecular pore that spans both membranes of coronavirus-induced DMV (18). Recently, Huang et al. presented a detailed model of SARS-CoV-2 DMV molecular pore obtained from cryo-ET (9). The enormous pore complex is composed of 12 copies each of nsp3 and nsp4 and consists of several distinct layers: a cytosolic crown formed by nsp3 (Fig. 1C), a belt within the DMV lumen comprising interconnected nsp3 and nsp4 ectodomains; transmembrane segments of nsp3 and nsp4 that anchor the pore within the highly curved DMV membrane; and, finally, an opening toward the DMV inner space formed by nsp4 C-terminal Endo domains. The crown is organized into layers with distinct symmetries: the lower base is composed of two concentric hexameric rings formed by the C-terminal region of nsp3, while the upper base and prongs are formed by six N-terminal regions of nsp3 (Fig. 1C).

Nsp3 (which corresponds to nsp2 in arteriviruses) is the largest non-structural protein and comprises between 10 and 16 domains depending on the virus genus (19). Figure 1A depicts the domain organization of SARS-CoV-2 nsp3. The protein can be partitioned into two distinct sections: the structurally and functionally well-characterized first two-thirds (nsp3N), and the less well-studied membrane-bound C-terminal section (nsp3C). A Ubiquitin-like domain 1 (Ubl1), hypervariable region (HVR), a macrodomain 1 (Mac1), PL2pro protease, and its accessory Ubl2 domain from the N-terminal part of nsp3 are present in all coronaviruses, while other domains vary among coronaviruses from different genera (19, 20).

The C-terminal region of nsp3 comprises two transmembrane regions, TM1 and TM2, with an ectodomain (3Ecto) in between, followed by the cytoplasmic Y region, which consists of Y1 and CoV-Y domains (19). The length of TM1 suggests a single membrane-spanning α-helix, whereas the topology of TM2 remained unclear until recently. Immediately following TM2 is the Y1 domain (Fig. 1A), which has also been bioinformatically identified in toroviruses and bafiniviruses of the *Tobaniviridae* family due to the presence of two distinctively conserved, potential Zn-binding motifs (Zn-BM1 and Zn-BM2) (21). Furthermore, a domain featuring a single His/Cys cluster was predicted to be present in the *Arteriviridae* family (19). Currently, no experimental structure of this domain at atomic resolution is available for any virus, and the only structural information for the SARS-CoV-2 Y1 domain comes from AlphaFold (22) modeling (9, 23).

The CoV-Y domain, which immediately follows Y1 and comprises the last 369 residues of SARS-CoV-2 nsp3, is believed to be exclusive to coronaviruses. A recently determined X-ray structure (23) revealed that it contains three subdomains (Y2, Y3, and Y4) with distinctive topologies. The Y1, Y2, and Y4 subdomains were found to have novel folds, with no analogous structures found in the Protein Data Bank (PDB). This poses a challenge in deducing their functions, as they lack structural resemblance to proteins with established functionalities. Although the monomeric structure of CoV-Y and the full Y region resolved in the DMV molecular pore contain the same subdomains, they adopt markedly different overall architectures (Fig. 1D). In the crystal structure, the three subdomains form a compact globular assembly. In contrast, within the pore, paired Y1/Y2 and Y3/Y4 subdomains are extended: hexameric Y1/Y2 units form the inner channel of the cytosolic crown, whereas Y4 extends upward to interact with the Ubl2-PL2pro region of nsp3N, thereby linking the upper and lower parts of the crown (Fig. 1C).

Posited by the cryo-EM structure of the DMV pore, the N-terminal portion of Y1 immediately following TM2 and containing the first Zn-binding motif (Zn-BM1) is extended. In this configuration, Zn(II) coordination is not possible because the predicted ligands are spatially separated (Fig. 1D). However, the Zn-binding residues are 100% conserved across all coronaviruses, suggesting that Zn(II) association plays an important role outside of the assembled DMV pore, possibly during the early stages of membrane remodeling and pore biogenesis. However, the functional role of either of the two Zn(II)-binding motifs has not yet been established.

In this study, we combined AlphaFold structure prediction, structure-informed multiple sequence alignments, and atomistic molecular dynamics simulations to investigate the structure and conservation of the TM2-Y region of nsp3 across the *Nidovirales* order. Challenging earlier views that the CoV-Y domain is unique to coronaviruses, we show that the TM2-Y region is present in most vertebrate-infecting nidoviruses. Furthermore, our analyses reveal that the overall topology, including the uniquely shaped TM2 segment, the Zn-binding Y1 domain, its adjacent Y2 domain, and Y4-like structural elements, is remarkably conserved despite extreme sequence divergence. The preserved structural scaffold suggests a shared mechanism for anchoring and stabilizing DMV pores within highly curved membranes. Furthermore, the universally conserved Zn-binding motifs likely mediate early membrane remodeling events during DMV biogenesis.

## RESULTS

### Experimental approach

To investigate the structure and dynamics of nidoviral TM2 and Y domains in the membrane environment, we employed a combination of multiple sequence alignments (MSAs), protein structure modeling, and molecular dynamics simulations (MD). We first focused on the SARS-CoV-2 nsp3 C-terminal region, encompassing both TM2 and all four Y subdomains (residues 1,494–1,945), and predicted the structure using AlphaFold 3 (AF3) (24). Taking advantage of the AF3 ability to model metal ions, we incorporated two Zn(II) ions, allowing characterization of the geometry of two putative Zn-binding motifs in the Y1 subdomain. We deliberately excluded TM1 from our modeling, as the current AF3 algorithm does not explicitly account for the membrane environment, which can significantly influence the packing of transmembrane helices. Following the initial structure prediction, we selected the top-ranked AF3 model for further analysis and performed MD simulations in a lipid bilayer to (i) determine TM2 positioning in the membrane, (ii) monitor rearrangement among the Y subdomains, and (iii) assess membrane-induced effects on the entire TM2-Y region. To more accurately model TM2 orientation in the membrane, the bilayer was composed of POPC (1-palmitoyl-2-oleoyl-sn-glycero-3-phosphocholine) and POPG (1-palmitoyl-2-oleoyl-sn-glycero-3-phosphoglycerol) lipids at a 9:1 molar ratio. POPC, a naturally abundant diacylglycerol phospholipid, was selected as the primary component due to its abundance in the ER membrane (25). POPG, a negatively charged phospholipid widely used to probe membrane-protein interactions, was added to introduce negatively charged headgroups (for details, see Materials and Methods).

To extend our analysis to other nidoviruses, we initially used AlphaFold 2 (22) to search for TM2-Y regions in representative viruses from all families within the order *Nidovirales*. This approach allowed the identification of Y1 and Y2 subdomains, as well as Y4-like regions, in *Tobaniviridae, Gresnaviridae*, *Olifoviridae,* and *Arteriviridae*. For all viruses, the TM2-Y regions were detected; we then constructed MSAs. These alignments were manually curated using the predicted structural information, and the final structural models of representative species from each group were calculated using the AF3 server. We further performed MD simulations in a lipid bilayer for representative viruses from both families, which provided insights into potential roles of these conserved regions in the viral life cycle.

### Topology of TM2 from SARS-CoV-2

**Fig 2:**
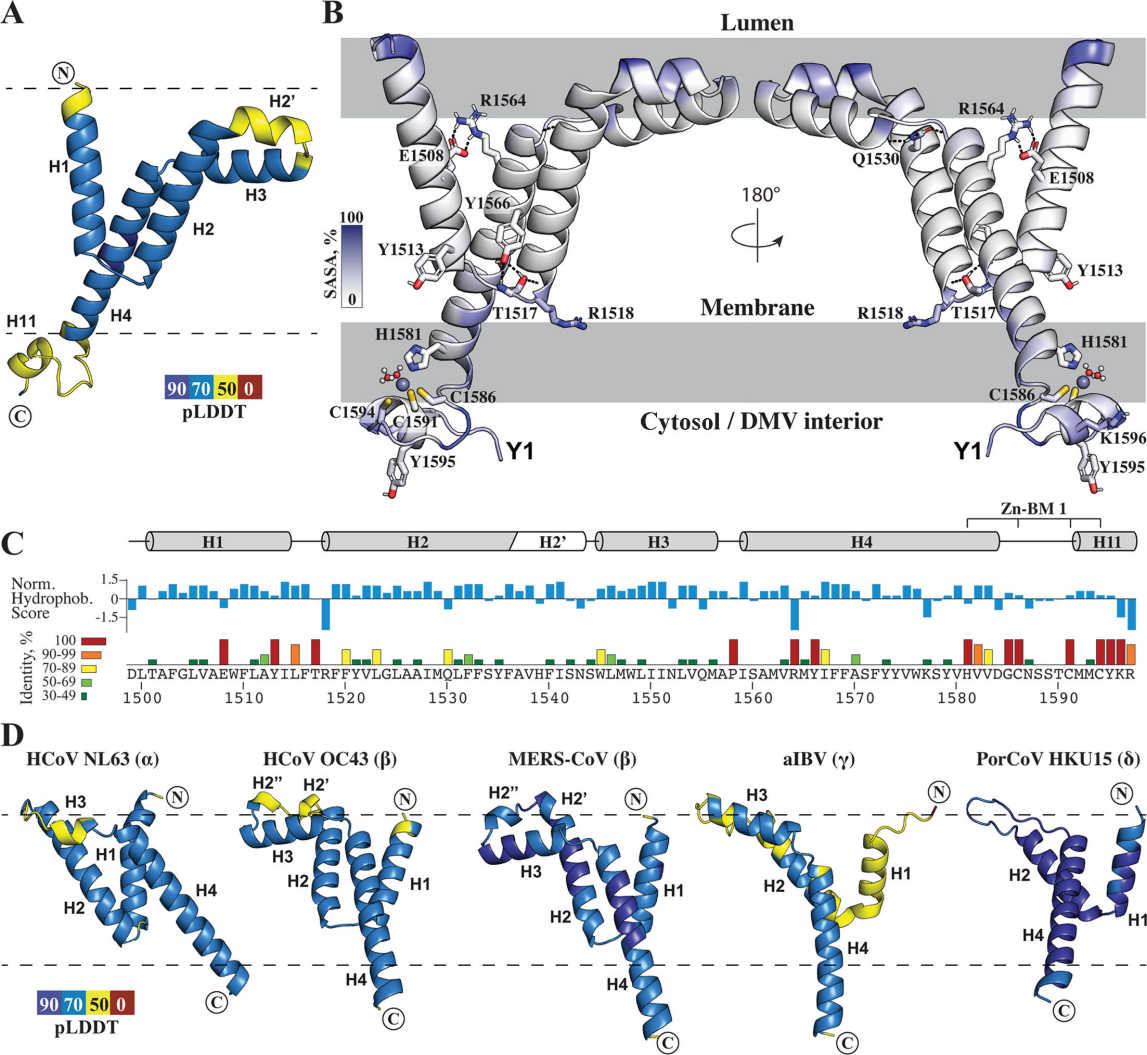
Transmembrane region TM2 in coronaviruses. (**A**) AlphaFold model of SARS-CoV-2 TM2 colored by pLDDT scores, used as the starting point for MD simulations. (**B**) Structure of SARS-CoV-2 TM2 after 500 ns of MD simulation in a full-atom lipid bilayer. The ribbon is colored by per-residue solvent-accessible surface area (SASA), and the approximate positions of lipid headgroups are indicated by gray blocks. Side chains of residues that are 100% conserved across betacoronaviruses are shown with sticks and labeled. (**C**) Sequence of SARS-CoV-2 TM2 showing per-residue normalized hydrophobicity and sequence conservation represented as identity scores computed across all betacoronaviruses. Helical segments are indicated by cylinders above the sequence. (**D**) AlphaFold models of nsp3 TM2 from selected coronaviruses colored by pLDDT scores. Dashed lines indicate the membrane boundaries predicted by PPM 3.0. server.

Previous studies have shown that the N-terminus of TM2 faces the ER lumen, whereas the C-terminus faces the cytoplasm; however, the overall topology of TM2 remained unclear until recently (26, 27). This hydrophobic 77-residue-long region (residues 1500–1576 of SARS-CoV-2 nsp3) is too long for a single transmembrane pass; however, it contains charged and polar residues that prevent the formation of three fully spanning membrane helices (Fig. 2C). The cryo-EM structure has provided insights into the 12-mer arrangement of TM2 within the DMV molecular pore (9). Here, we investigated the structure of monomeric TM2-Y embedded in a flat membrane, representing its conformation during early stages of membrane remodeling before DMVs are formed.

Figure 2A illustrates the structure of the TM2 domain predicted with AlphaFold and colored by predicted Local Distance Difference Test (pLDDT) scores. Most of TM2 is predicted with pLDDT >70, indicating high confidence in the local backbone conformation. Peripheral regions were predicted less reliably (50 < pLDDT < 70), likely reflecting the requirement for a constraining membrane environment for proper folding. To characterize TM2 structure in a membrane environment, we performed MD simulations in a full-atom lipid bilayer using AlphaFold model as the starting point. Figure 2B depicts the TM2 domain after a 500 ns MD simulation. Within the flat membrane, TM2 adopts a unique topology consisting of four α-helices (H1–H4), of which only one (H4) fully spans the membrane. The first two α-helices, H1 (residues 1496–1515) and H2 (residues 1518–1543), form a hairpin that is embedded within the membrane but does not traverse it. The tip of the hairpin is anchored to the cytosolic leaflet lipid headgroups via positively charged R1518; however, this residue is not conserved beyond the closest relatives of SARS-CoV-2 (Fig. S1). H2 is six residues longer than H1 and features a kink at residue A1537, bending the helix by ~70° to form a distinct segment, H2’. Together with H3 (residues 1545–1556), H2’ is embedded in the luminal side of the membrane. Notably, H3 is buried deeper within the lipid bilayer. Both H2’ and H3 exhibit amphipathic properties, with their luminal-facing sides enriched in polar residues and their membrane-facing sides predominantly hydrophobic. This is illustrated in Fig. 2B, which depicts the TM2 structure colored according to per-residue solvent-accessible surface area (SASA) calculated in PyMOL, and Fig. 2C, which shows the hydrophobicity score calculated using the ProtScale tool (28) on web.expasy.org website (29).

The fourth α-helix, H4 (residues 1559–1584), is the longest and spans the entire membrane bilayer. H4 is packed along H2, forming three turns of hydrophobic contacts, with the α-helices aligned in parallel but shifted relative to each other along the axis perpendicular to the membrane plane (Fig. 2B). The polar residue Q1530, located in the middle of H2, forms hydrogen bonds with the backbone of the region between H3 and H4, specifically residues V1554, A1557, and I1559. Q1530 is 73% conserved among betacoronaviruses and 100% conserved in alphacoronaviruses. H4 is connected to H1–H2 via two distinct interactions: a salt bridge between E1508 (H1) and R1564 (H4) and a hydrogen bond between T1517 (H1–H2 hairpin tip) and Y1566 (H4). Both contacts are 100% conserved in betacoronaviruses, suggesting their relevance for maintenance of overall TM2 arrangement (Fig. S1A).

Overall, TM2 adopts a wedge-like shape, with a wider luminal side characterized by the amphipathic helices H2’ and H3, which narrows toward the opposite side of the membrane. This shape is well-suited for curved membranes, as observed experimentally in the cryo-EM structure of the DMV molecular pore, where the DMV membrane has a round cross-section (9).

### TM2 topology is conserved across coronaviruses

Given the structural role of TM2 in anchoring nsp3 to highly curved membranes, we hypothesized that the overall topology should be conserved across coronaviruses despite relatively low sequence similarity. The overall amino residue identity score for SARS-CoV-2 TM2 among betacoronaviruses varies from 25% to 44%, except for the closest homolog of SARS-CoV-2, SARS-CoV, with an identity score of 79.5%. Comparison across the four coronavirus genera reveals even greater sequence diversity. The low sequence similarity complicates multiple sequence alignment; for instance, BLAST (30) fails to detect significant similarity between SARS-CoV-2 TM2 and TM2 sequences from more distantly related gamma- and deltacoronaviruses. Thus, to compare TM2 topology among various coronaviruses, we aligned TM2 sequences of each genus separately, using 20 α-, 15 β-, 5 γ-, and 7 δ-CoVs (Fig. S1A, Table S1) and then modeled TM2 structures for several representatives from each group using AlphaFold (Fig. 2D ; Fig. S1B). The orientation of TM2s in lipid bilayer was calculated by PPM 3.0 server (31). Comparative analysis of the models revealed that all coronaviruses share the same wedge-like TM2 structure, consisting of the helical hairpin (H1 and H2) embedded in the membrane, followed by an amphipathic region (H2’ and H3 in SARS-CoV-2), and ending in a long membrane-spanning α-helix, H4.

The main difference in TM2 topology between different genera of coronaviruses is in the amphipathic region between H2 and H4 (Fig. 2D ; Fig. S1B). In betacoronaviruses*,* α-helix H3 is consistently present, while the region corresponding to H2’ in SARS-CoV-2 varies; it may be shorter, fully unstructured, or contain a separate short α-helix. In gammacoronaviruses, H2’ is absent, while an H3 is fully formed and is 13 residues long. Deltacoronaviruses have a 13-residue mostly hydrophobic loop between H2 and H4, whereas alphacoronaviruses preserve the same loop length but contain a short 4-residue α-helix beginning with a conserved tryptophan residue, resembling H3 in SARS-CoV-2.

Overall, despite sequence variation, TM2 from all coronavirus genera maintains the same wedge-shaped architecture, with a broad luminal face formed by the H1–H2 hairpin and amphipathic α-helices, narrowing to the C-terminal end of the transmembrane α-helix H4.

### Isolated coronaviral Nsp3 Y adopts a compact conformation

Previously, we used AlphaFold to model the Y region of human alpha- and betacoronaviruses (23). In this work, we extended our analysis to other coronaviruses, including gamma- and deltacoronaviruses. We aligned nsp3 Y sequences for 20 α-, 15 β-, 5 γ-, and 7 δ-CoVs (Table S1 and Fig. S2 through S4) and then curated these alignments using AlphaFold structural models for several representatives of each subfamily.

**Fig 3:**
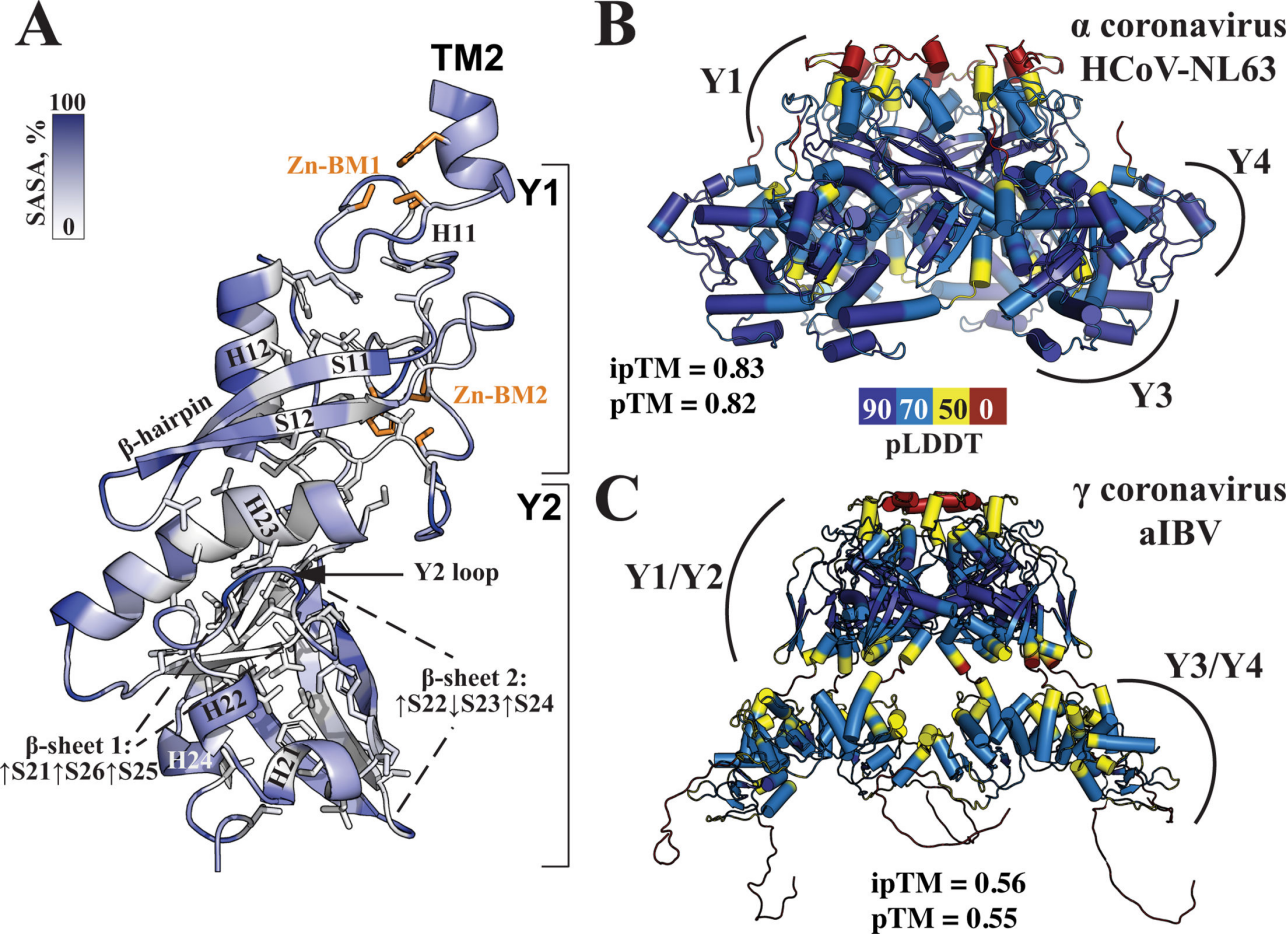
Conservation of Y1/Y2 tandem and its oligomerization mechanism among coronaviruses. (**A**) AlphaFold model of the SARS-CoV-2 nsp3 Y1-Y2 region shown as a ribbon and colored by per-residue solvent-accessible surface area. Residues comprising the hydrophobic core (SASA <10%) are displayed as sticks. (**B and C**) AlphaFold-predicted hexamers from the α coronavirus HCoV-NL63, and the γ coronavirus aIBV, colored by pLDDT scores. Confidence scores, ipTM and pTM, are indicated for each model.

Across all predicted structural models, the individual domain topologies remained consistent, except for Y3, which forms a four-helix bundle in betacoronaviruses but a three-helix bundle in all other genera. In all models, the Y1 and Y2 subdomains form a stable tandem association mediated by a shared hydrophobic core centered around a long α-helix (H23 from Y2). Figure 3A shows the structure model of the SARS-CoV-2 Y1 and Y2 domains, colored by solvent-accessible surface area, side chains of residues forming the hydrophobic core shown with white sticks (SASA <10%). Within the Y1 subdomain, the hydrophobic core includes a β-hairpin (S11/S12) resting on H23, the hydrophobic side of an α-helix (H13), and elongated unstructured loops stabilized by the second Zn-binding motif (Zn-BM2). This hydrophobic core extends into the Y2 subdomain through a β-sandwich, which is externally enclosed by short helices and loops. This structural model is supported by our experimental findings, which suggest that Y1 requires Y2 for structural stability. In particular, we were unable to obtain soluble nsp3 Y1 domain in isolation while purification of SARS-CoV-2 nsp3 CoV-Y (Y2-Y4) was successful (23, 32).

Although structural modeling of the nsp3 Y region across different coronavirus genera confirmed the topologies of all four Y subdomains, the overall shape of the Y region varied. In alpha- and betacoronaviruses, including SARS-CoV-2, the Y region adopts a closely packed globular structure, characterized by tight inter-subdomain interactions: Y1/Y2, Y2/Y3, and Y3/Y4 (Fig. 3B; Fig. S5A). This compact shape resembles that observed in the X-ray structure of SARS-CoV-2 CoV-Y (23). On the other hand, the Y region in gamma- and deltacoronaviruses adopts a more extended conformation, retaining only Y1/Y2 and Y3/Y4 interactions (Fig. 3C; Fig. S5C). As a result, hexamers for all tested gamma- and deltacoronaviruses exhibit lower pTM and ipTM scores (0.5–0.6) compared with those for alpha- and betacoronaviruses (>0.8). These reduced scores likely reflect the loss of association of the Y3/Y4 globules with both Y1/Y2 and neighboring Y3/Y4 subunits. This interpretation is supported by the observation that, for the shortened aIBV hexamer, containing only Y1/Y2, both pTM and ipTM scores increase above 0.8, indicating a reliable complex prediction (Fig. S5D). Notably, in the cryo-EM model, SARS-CoV-2 nsp3 Y is in an extended conformation in the DMV pore (9), similar to gamma- and deltacoronaviruses. Nonetheless, the conservation of a compact structure in alpha- and betacoronaviruses suggests a potential function for the globular conformation.

To investigate the conformational dynamics of nsp3 Y and ascertain whether nsp3 Y domain adopts an extended conformation in the absence of other DMV pore components, we performed MD simulations in a membrane bilayer. During the 500 ns simulation, the Y subdomains maintained a compact fold throughout the entire trajectory, with the radius of gyration for nsp3 Y (residues 1592–1940) measuring at 2.24 ± 0.08 nm (Fig. S6A). Distances between individual subdomains, and between the Y region and the membrane bilayer, remained stable (Fig. S6B). Over the entire simulation, we did not observe any tendency for nsp3 Y to adopt the extended subdomain arrangement observed in the cryo-EM molecular pore structure or other noticeable domain motions.

Although 500 ns is insufficient to conclude that the compact form is the sole conformation outside the assembled DMV pore complex, our results suggest that the compact structure observed for SARS-CoV-2 nsp3 CoV-Y (23) is not a result of crystal packing. This conformation appears to be sampled in solution and may represent a functionally relevant state.

### Zn-binding sites in Y1 and their interactions with solvent

Coronaviruses possess two Zn-binding His/Cys motifs within Y1, Zn-BM1, and Zn-BM2. These residues are 100% conserved (Fig. S2) and have been used in previous studies as bioinformatic markers to identify Y1-like domains in other nidoviruses (19). However, the precise function of these motifs remains unknown.

**Fig 4:**
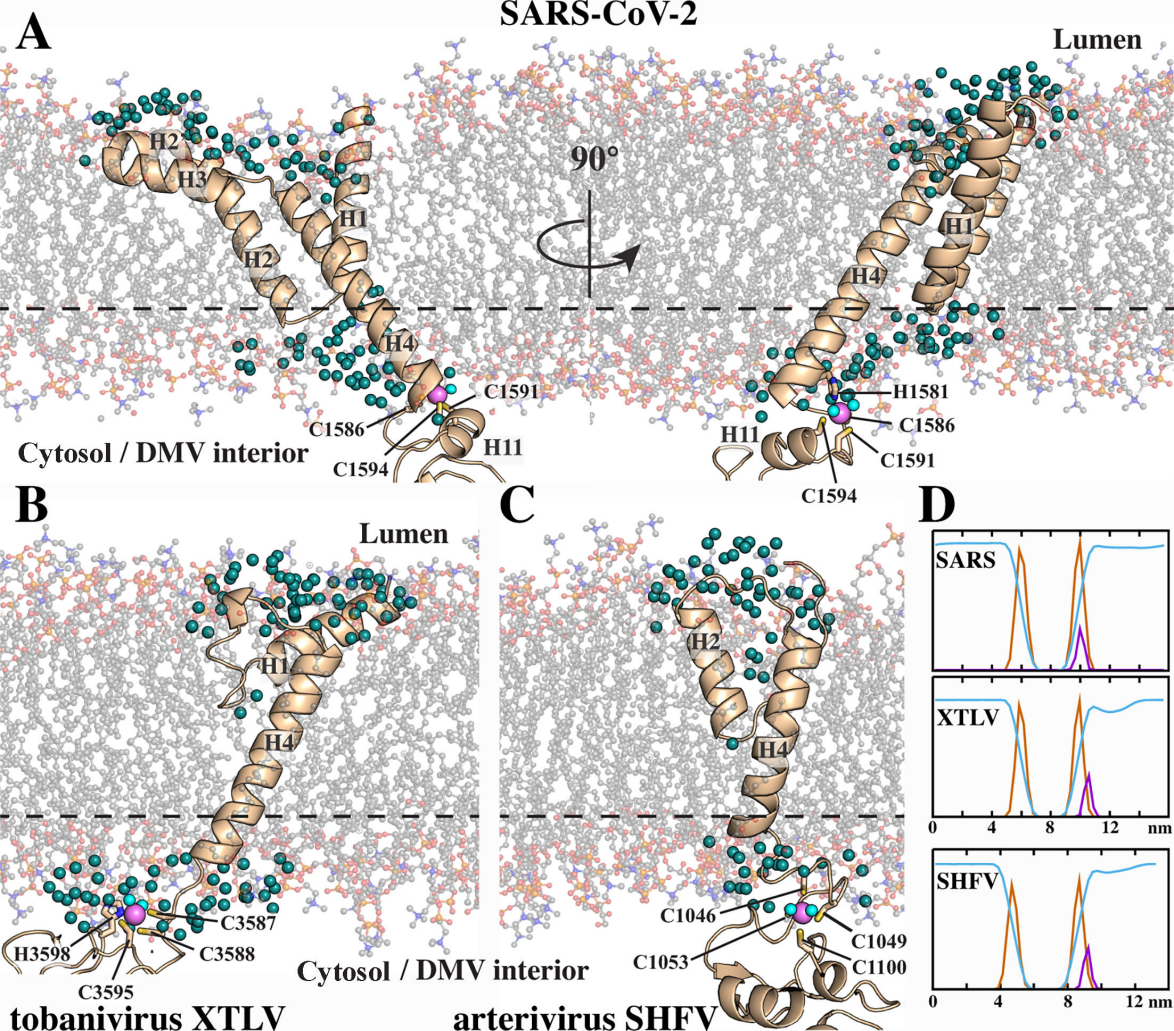
Molecular dynamics simulations of the TM2-Y region from SARS-CoV-2 and the corresponding regions in representative tobani- and arteriviruses. (**A–C**) Final structures after 500 ns MD simulation in a full-atom lipid bilayer. Lipid heavy atoms are shown as ball-and-stick models, and the proteins are shown as ribbons. Zn(II) ions are colored in magenta, and metal-coordinating residues are shown as sticks and labeled. Water molecules (oxygen atoms) within a 3.5 Å radius of the hydrophobic region of TM2 are shown as teal spheres, while the two water molecules participating in Zn(II) octahedral coordination are shown in cyan. The dashed line indicates the boundary of the membrane hydrophobic core. (**D**) Distribution along the Z-axis of water molecules (light blue), phosphorus atoms from POPC and POPG lipids (orange), and Zn(II) ions (magenta) over the final 100 ns of the MD trajectories. Distributions were normalized to a common scale to account for differences in the number of atoms within each group.

In the AlphaFold model of SARS-CoV-2 nsp3 Y, Zn-BM1 coordinates Zn(II) ion by H1581 from the C-terminal turn of the last transmembrane α-helix (H4), C1594 from the first α-helix of Y1 (H11), and two residues, C1586 and C1591, located in the loop between these helices. This coordination geometry tethers the five-residue α-helix H11 of Y1 toward the membrane surface (Fig. 4A), and positions the Zn(II) ion within the membrane headgroup layer during the MD simulations. As shown in Fig. 4D (top), density profiles of lipid phosphorus atoms (orange) and the Zn(II) ion (magenta) calculated over the final 100 ns of the trajectory exhibit overlapping peaks, indicating that the Zn(II) ion resides at the depth of the phosphate groups within the bilayer.

Zn-BM2 coordinates Zn(II) through four residues, C1627, H1630, C1634, and C1637, located within a 30-residue-long unstructured region between Y1 β-hairpin and α-helix H12. Zn(II) appears to have a structural role, stabilizing the Y1 hydrophobic core by restricting the position of the loop between the Y1 β-hairpin and α-helix H13 (Fig. 3A). Although Zn-BM2 acts as a structural element, it is solvent-exposed and was predicted by AlphaFold to adopt an octahedral (C6) coordination geometry, in which two additional water molecules coordinate the Zn(II) ion. Such octahedral coordination is uncommon for protein-associated Zn(II) ions, particularly in structural Zn-binding motifs (33).

To assess the solvent accessibility of the two Zn-binding motifs, we monitored interactions of these regions with water during the MD simulations. Zn-BM2 consistently maintained octahedral coordination throughout the trajectory, with an average Zn-O(water) distance of 2.11 ± 0.05 Å and 6–8 water molecules within a 3.5 Å radius at all times. Despite its solvent exposure, Zn-BM2 maintained a stable local structure. Conversely, Zn-BM1, initially modeled in a tetrahedral (C4) conformation, transitioned to an octahedral geometry after approximately 7 ns of the simulation, accompanied by a distinct change in ligand-Zn(II) angles to 90, 90, and 180 degrees (Fig. S6C). Similar to Zn-BM2, the minimum Zn-O(water) distance remained 2.11 ± 0.05 Å. The number of molecules in the second hydration shell (within 3.5 Å of Zn(II)) increased from 3 to 6–7, occasionally fluctuating to 8, after the coordination geometry transition. The presence of water molecules in both Zn-BMs suggests an environment more typical of solvent-exposed catalytic Zn-binding sites rather than classical structural Zn-binding motifs (34).

In addition to water coordination by the two Zn-binding motifs, we also observed penetration of water molecules into the membrane in the vicinity of Zn-BM1. Figure 4A shows water molecules within 3.5 Å of the TM2 hydrophobic region, located inside the POPC/POPG layer and reaching the tip of H1–H2 hairpin. We hypothesize that this effect is promoted not only by the presence of Zn(II) ion but also by the unique amino acid composition of TM2. Specifically, the side of the H4 α-helix facing the H1–H2 hairpin contains several polar residues (Fig. 2C). Consequently, the presence of water molecules near these residues is not energetically unfavorable. The last three turns of H4 and the tip of the H1–H2 hairpin are partially solvent-exposed, with SASA exceeding 10% (Fig. 2B). Across all coronaviruses, multiple charged or polar residues are consistently found in the C-terminal half of H4 (Figs. S1 and S2). Despite low overall sequence similarity, this uniform enrichment in hydrophilic residues within a transmembrane helix likely reflects an evolutionary adaptation facilitating local water penetration into the membrane.

### Conservation of nsp3 TM2-Y domains in nidoviruses infecting vertebrates

Replicative organelles such as DMVs are a common feature of nidoviruses. Thus, it is reasonable to suggest that the recently discovered SARS-CoV-2 molecular pore complex, which connects DMV interior to the cytoplasm, is also conserved beyond coronaviruses. Previous bioinformatic studies suggested that the CoV-Y region of nsp3, consisting of subdomains Y2, Y3, and Y4, is exclusive to coronaviruses (19). Based on the observation that the Y1 domain intimately interacts with Y2 and that these two domains form the inner channel of the DMV pore crown (9), we hypothesized that at a minimum, the Y1/Y2 tandem should be conserved beyond coronaviruses. Identifying corresponding regions within the polyproteins of less studied nidoviruses is challenging since domain annotation of large segments of pp1a/b is lacking for many viruses due to very low sequential similarity. Nonetheless, the combination of various bioinformatics approaches previously identified the putative Y1 domain in *Tobaniviridae* and *Arteriviridae* families of order *Nidovirales*. Furthermore, some *Tobaniviridae* genera were proposed to have the entire Y region (21).

We leveraged AlphaFold to identify and structurally analyze putative Y regions in representative viruses from all viral families within the order of *Nidovirales* (Table S1). Our analysis revealed a region resembling coronavirus nsp3 Y in four nidoviral families beyond *Coronaviridae: Tobaniviridae* and three families from suborder *Arnidovirineae — Gresnaviridae*, *Olifoviridae,* and *Arteriviridae* (for clarity, we will refer to the latter three as arteriviruses, as *Arteriviridae* is the most numerous family). Notably, all these viruses infect vertebrates and have been shown to promote the formation of DMVs during the infection (35, 36). In contrast, three other vertebrate-infecting nidoviral families (*Cremegaviridae, Nanghoshaviridae,* and *Nanhypoviridae*), as well as all six families infecting invertebrates, lack nsp3 Y, including the two characteristic Zn-BMs in Y1 subdomain.

**Fig 5:**
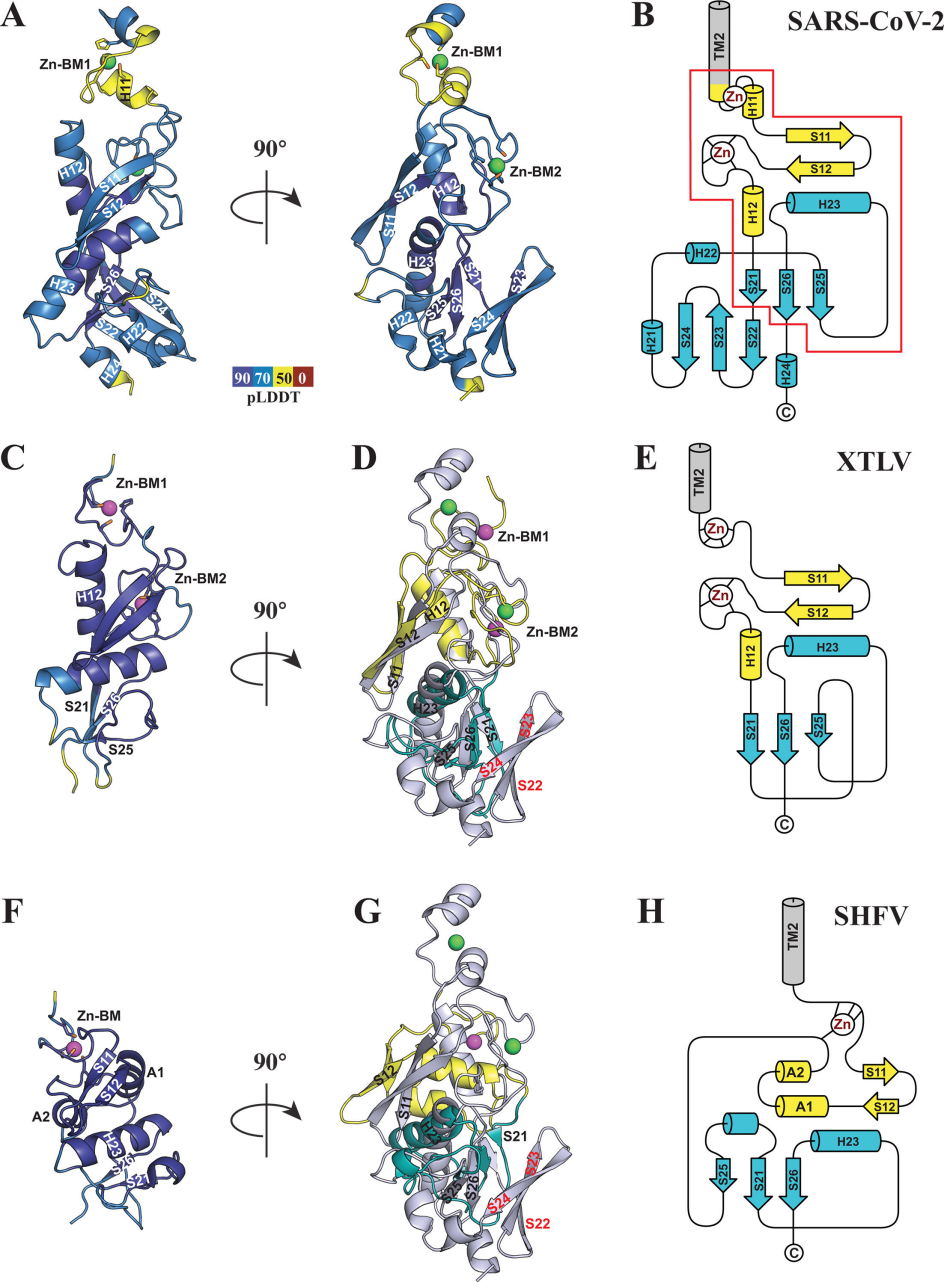
AlphaFold models of Y1/Y2 subdomains and their topology in representative nidoviruses. (**A, C, F**) Structural models for the coronavirus SARS-CoV-2 (**A**), tobanivirus XTLV (**C**), and arterivirus SHFV (**F**), colored by pLDDT scores. Zn(II) ions are shown as spheres, and side chains of residues forming the 100% conserved Zn-binding motifs are displayed as sticks. (**D and G**) Structural overlays of Y1/Y2 subdomains from XTLV (**D**) and SHFV (**G**) with Y1/Y2 subdomains from SARS-CoV-2. The SARS-CoV-2 Y1/Y2 tandem is colored in gray with Zn(II) ions shown in green, whereas Y1, Y2, and Zn(II) from XTLV and SHFV are colored yellow, teal, and magenta, respectively. The β-strands forming an additional sheet unique to coronaviruses are marked with red labels. (**B, E, H**) Topology diagrams of Y1/Y2 subdomains for SARS-CoV-2 (**B**), XTLV (**E**), and SHFV (**H**). The minimal conserved Y1/Y2 fold shared among all three viruses is indicated by a red outline in panel B. Secondary structure elements in XTLV and SHFV are labeled according to their SARS-CoV-2 equivalents for comparison.

In all instances where a Y-like region was identified, it is consistently positioned between TM2 of nsp3 (or nsp2 in arteriviruses) and the beginning of nsp4 (nsp3 in arteriviruses), as expected. Similar to the coronaviruses, the Y domains of tobaniviruses and arteriviruses contain Y1 and Y2 subdomains, which intimately interact with each other (Fig. 5C and F). The rest of nsp3 Y in these viruses differs significantly from its counterpart in coronaviruses. First, they lack the Y3 subdomain. Second, the C-terminal segment does not fold into the well-formed Y4 subdomain. However, it contains structural elements similar to those found in the Y4 of coronaviruses (discussed in detail below).

### Y1 domain

Y1 from Tobaniviridae closely resembles the Y1 topology observed in coronaviruses. Panels C and E in Fig. 5 depict the AlphaFold-predicted structure colored by pLDDT confidence scores and the topology of the Y1/Y2 tandem from Xinzhou toro-like virus (XTLV), respectively. The Y1 domain of tobaniviruses contains both Zn-BM1 and Zn-BM2; however, their compositions differ from those found in coronaviruses. Specifically, the first Zn-BM1 is the CCCH type, and the second is the CHCH type, as opposed to HCCC and CHCC types found in coronaviruses (Fig. S7). Despite these differences, the overall positioning of the Zn-BM1 and Zn-BM2, as well as the involvement of Zn-BM2 in maintaining the Y1 hydrophobic core, remains consistent between tobaniviruses and coronaviruses (Fig. 5D).

Previous bioinformatic studies predicted the presence of a Y1-like domain in nsp2 of arteriviruses (21). The domain is much smaller, about 60 residues against ~80 in tobaniviruses and ~90 in coronaviruses, and contains only one potential Zn-binding motif. We studied 15 arteriviruses (Table S1) and found that despite apparent differences, the overall Y1 topologies of arteriviruses and coronaviruses share many similarities, including a β-hairpin in Y1 packed against a long α-helix in Y2 (Fig. 5F). Figure 5H shows the topology of the modeled Y1-like domain of nsp2 from Simian Hemorrhagic Fever Virus (SHFV). The only Zn-BM of arteriviruses is of the CCCC type, with the exception of the GGGSV virus, which contains Zn-BM of the HCCH type (Fig. S8). Three cysteines of the Zn-BM are from the N-terminal part of the domain, where Zn-BM1 is typically located in coronaviruses. The fourth cysteine is from the loop situated between α-helix A2 and β-strand S21 (Fig. 5F). The positioning of the Zn-BM is similar to that of Zn-BM1 in coronaviruses, and it appears to stabilize the conformation of the N-terminus (Fig. 5G). While coronaviruses utilize the second Zn-BM to stabilize the hydrophobic core, in arteriviruses, the core is formed by residues from two nearly parallel helices, A1 and A2, unique to arteriviruses.

### Y2 domain

The Y2 domain in tobaniviruses and arteriviruses is notably smaller, comprising approximately 50–60 residues, in contrast to the larger Y2 of coronaviruses, which ranges from 88 to 97 residues (Fig. S3). This smaller size is primarily due to the absence of the β-sheet comprised of S22, S23, and S24 in coronaviruses. The remaining parallel β-sheet in tobani- and arteriviruses corresponds to the S21, S25, and S26 β-strands in coronaviruses (Fig. 5E and G). Like in coronaviruses, it contributes to the formation of the Y2 hydrophobic core along with the long H23 α-helix and is similarly positioned.

Y2 is the least conserved domain in *Tobaniviridae,* with no residues exhibiting 100% identity (Fig. S7). This lack of conservation, despite structural similarity, explains why the presence of Y2 outside of the *Coronaviridae* family was not previously recognized. In arteriviruses, the Y2 domain also shows significant variability but nonetheless contains a few conserved residues. (Fig. S8).

In summary, we revealed that the Y1 domain, when present, is always accompanied by a Y2 domain. We identified a minimal Y1/Y2 tandem fold present in all five virus families, outlined in red in Fig. 5B. This fold consists of a long α-helix (H23) sandwiched between a parallel three-stranded β-sheet (Y2, lower part) and β-hairpin (Y1, upper part). The hydrophobic core is capped on top by one (or two in arteriviruses) α-helix and stabilized by Zn-binding loops.

### Y1/Y2 propensity for oligomerization is conserved beyond coronaviruses

**Fig 6:**
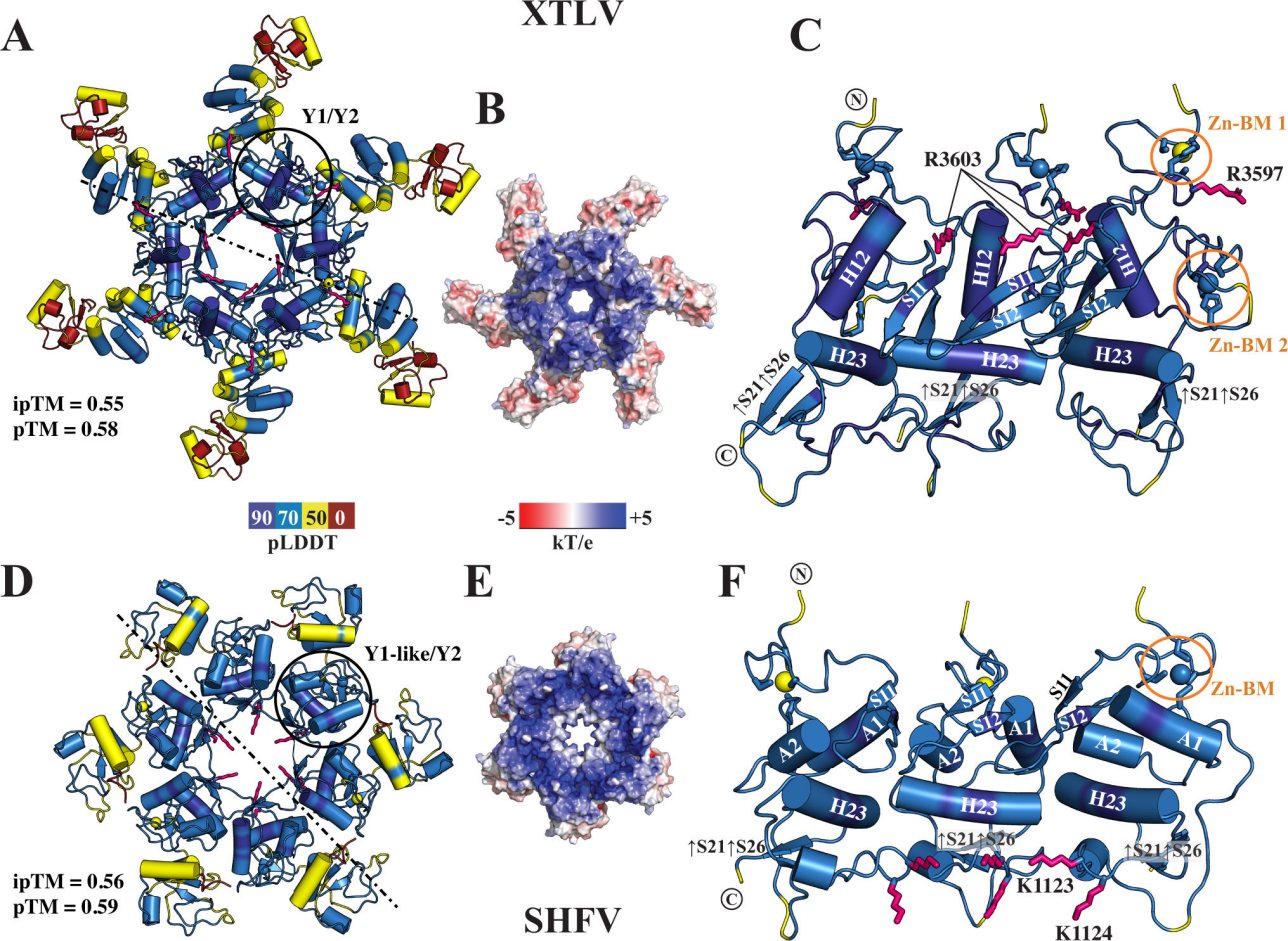
Y-like domains in tobaniviruses and arteriviruses form cyclic hexamers *in silico*. (**A and D**) AlphaFold models of the Y-domain hexamers from the tobanivirus XTLV (**A**) and arterivirus SHFV (**D**), colored by pLDDT scores. Confidence scores, ipTM and pTM, are indicated for each model. (**B and E**) The hexamers shown as surface representations and colored by electrostatic potential calculated using ABPS PyMOL plugin (38). (**C and F**) Cross-sectional cartoon representations of the Y1/Y2 hexamers shown in panels A and C, respectively. Secondary structure elements are labeled according to the topology presented in Fig. 5. The C-terminal regions following Y2 were omitted for clarity.

After confirming that a minimal Y1/Y2 fold found in coronaviruses is topologically conserved in tobaniviruses and arteriviruses, we hypothesized that Y1/Y2 tandems in these viruses could also form hexamers, similar to those observed in the SARS-CoV-2 DMV pore. To test this, we used AlphaFold-multimer (37) to evaluate the oligomerization propensity of Y regions from all four families in which they occur*— Tobaniviridae*, *Olifoviridae*, *Gresnaviridae,* and *Arteriviridae*. The modeling results showed that the viruses consistently form hexamers (Fig. 6A and D), with cyclic oligomers predicted for 12 out of 15 tested arteriviruses species and 7 out of 11 tobaniviruses species.

The overall architecture of the predicted Y-domain cyclic hexamers in tobaniviruses and arteriviruses closely resembles that of coronaviruses. The inner ring is formed by tightly packed Y1/Y2 domains, and a larger outer ring, consisting of loosely packed C-terminal regions. The oligomerization interface is conserved as well: the narrowest part of the central pore is lined by β-hairpins from the Y1 domain, while the long H23 α-helix from the Y2 domain contributes to the primary inter-subunit contacts (Fig. 6C and F), resembling the organization of Y1/Y2 hexamers in the SARS-CoV-2 DMV pore complex. The central pore measures approximately 1.35 nm in *Tobaniviridae* and 1.2 nm in arteriviruses*,* which is smaller compared to 1.6 nm in *Coronaviridae* (9).

Despite low sequence conservation, the electrostatic surface potential is preserved across all five viral families. Similar to coronaviruses (Fig. S5 (23)), both the membrane-facing side and the inner surface of the inner channel are positively charged in both tobani- and arteriviruses (Fig. 6B and E). For example, XTLV has two conserved positively charged residues: R3597 on the membrane-facing surface, and R3603 projecting into the pore (Fig. 6C; Fig. S7). In SHFV, the Y1-like domain contains N-terminal R1048 and R1055, and the pore-facing R1070. In coronaviruses, a long Y2 loop connecting α-helix H22 and the β-strand S25 points into the central channel of the DMV pore, where the positively charged residues, such as 100% conserved K1715 in SARS-CoV-2, contribute to the overall positive charge of the central channel of the DMV pore (Fig. S3). Similarly, the Y2 loop in arteriviruses contains two adjacent surface-exposed lysine residues, K1123 and K1124, in SFHV (Fig. 6F; Fig. S8). Furthermore, in *Tobaniviridae*, at least one lysine residue is present in the Y2 loop, although its exact position varies among different viruses.

Overall, these results suggest a model in which the positively charged upper surface of Y1 interacts with a membrane, while the positively charged pore interior facilitates the transport of newly synthesized vRNA. The conserved oligomerization mechanism and electrostatic profile across corona-, tobani-, and arteriviruses reinforce the hypothesis that the Y region is involved in pore formation in vertebrate-infecting nidoviruses that replicate in DMVs.

### Y4-like structural elements

**Fig 7:**
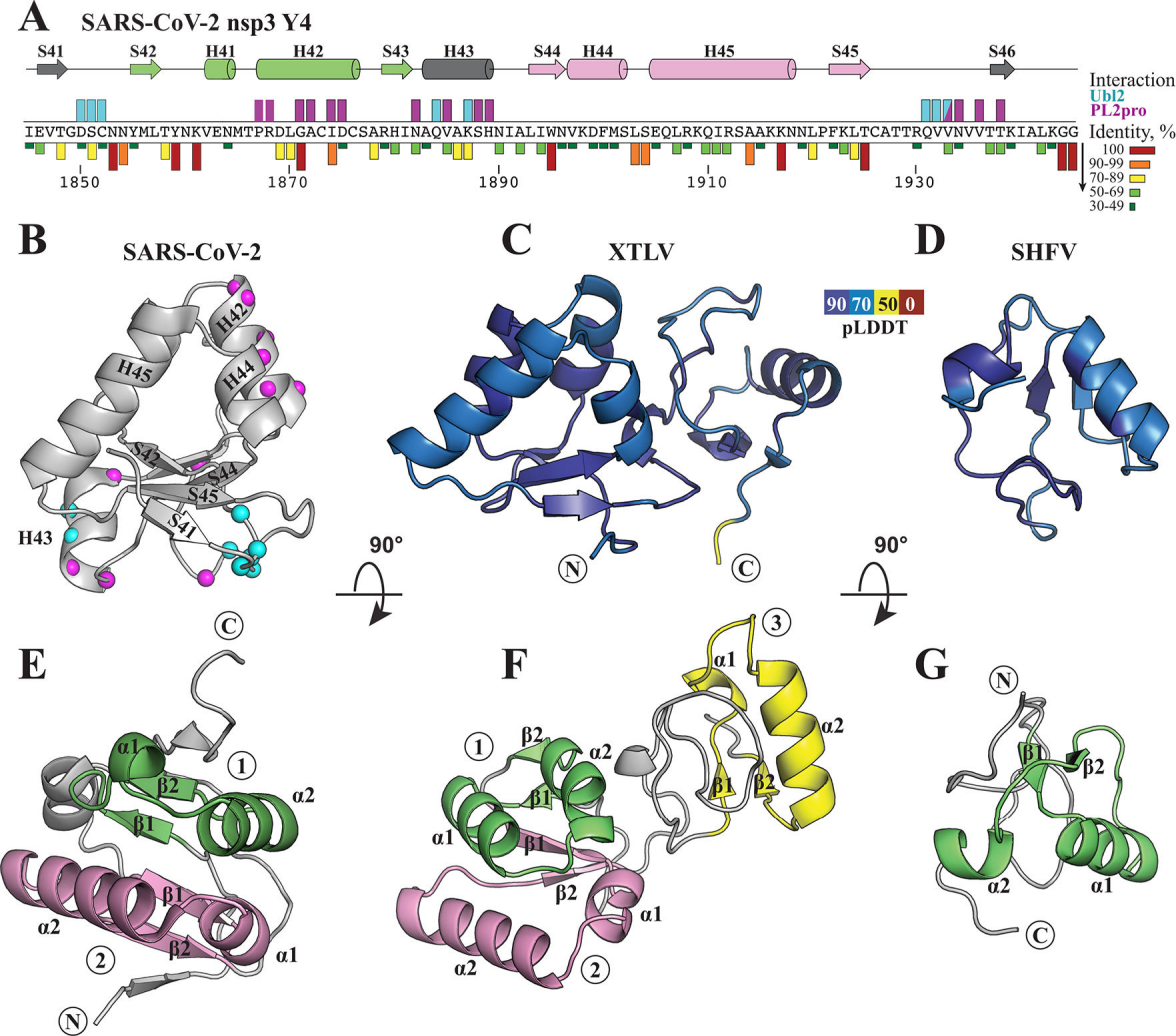
Topology of SARS-CoV-2 Y4 subdomain and structural conservation of βααβ elements in corresponding regions of tobani- and arteriviruses. (**A**) Sequence of the SARS-CoV-2 Y4 subdomain with secondary structure elements depicted at the top. Residues interacting with Ubl2 and PL2pro domains in the DMV pore are indicated above the sequence by cyan and purple bars, respectively. Sequence identity scores across all betacoronaviruses are shown below the sequence. (**B**) Crystal structure of the Y4 domain from SARS-CoV-2 (PDB 8F2E). C^α^ atoms of residues interacting with Ubl2/PL2pro in the DMV molecular pore are shown as spheres and colored as in panel A. (**C and D**) AlphaFold models of regions corresponding to SARS-CoV-2 Y4 in XTLV and SHFV, colored by pLDDT scores. (**E–G**) Structure of the Y4 domain from SARS-CoV-2 (**E**), and the models of the corresponding regions in XTLV (**F**) and SHFV (**G**) are colored according to βααβ repeats. The first repeat is in green, the second is in pink, and the third is in yellow.

In our previous determination of the structure of the SARS-CoV-2 CoV-Y domain, we were unable to identify any known domains with significant similarity to Y4 using the DALI server (23). In coronaviruses, the Y4 subdomain is a compact globular domain, which contains two 30-35 residue dome-like elements (referred to as “domes” below) of βααβ topology (Fig. 7A and B). In this arrangement, the two α-helices are positioned above a two-stranded parallel β-sheet, forming a triangular fold in which the β-sheet serves as the base and the helices form the sides (Fig. 7E). The compact fold of SARS-CoV-2 Y4 is stabilized by extensive hydrogen bonding between the two domes, with additional short β-strands from the N- and C-termini (S41 and S46) contributing to the overall β-sheet architecture (23). Y4 is the second most conserved subdomain after Y1 among the Y subdomains of coronaviruses (Fig. 7A; Fig. S4).

Although we did not identify a Y4 subdomain in tobani- and arteriviruses that resembles the coronavirus Y4, we consistently observed single or multiple βααβ elements in the C-terminal part of nsp3 (nsp2 in arteriviruses). AlphaFold predicts the presence of three domes in most tobaniviruses (Fig. 7C and F; Fig. S7). One exception is the torovirus genus (BEV and PoTV), where the region following Y2 is nearly three times shorter than in other tobaniviruses and shows poor sequence alignment with them (Fig. S7). For these two viruses, AlphaFold fails to predict a reliable secondary structure; however, the possibility that one dome forms cannot be excluded. In other tobaniviruses, structural models enable confident sequence alignment of the Y4-like region, although not all three βααβ elements are well predicted in some species. The first two domes are adjacent, separated by only a few residues, while the second and third domes are separated by a longer, mostly unstructured loop (Fig. 7F ; Fig. S7).

In arteriviruses, AlphaFold predicts a small globular domain between Y1/Y2 tandem and the C-terminus of nsp2 in the majority of tested species (12 out of 15) (Fig. 7D). For three viruses (EAV, HOFAV, and GGGSAV), AlphaFold could not predict a reliable globular structure, likely due to shallow multiple sequence alignments. In the remaining species, AlphaFold predicts a single dome covered on one side by a long, twisted loop (Fig. 7G; Fig. S8). This small globular domain is simpler than the SARS-CoV-2 Y4; however, the presence of a recurring βααβ element suggests that these folds are evolutionarily related and may perform similar functions.

The C-terminus of the nsp3 Y4 subdomain of coronaviruses and tobaniviruses is separated from the first transmembrane α-helix of nsp4 by only a few residues. In contrast, the Y4-like region of arteriviruses, with the exception of WAV and LDV, is followed by a long linker before the nsp2/nsp3 cleavage site (Fig. S8). The length of the linker varies from nine residues in RtClanAV to 44 in WPDV. For longer linkers, AlphaFold predicts the presence of at least one α-helix, albeit with a relatively low pLDDT score. Whether this arterivirus-specific region has a distinct structural or functional role remains to be determined.

In the SARS-CoV-2 DMV molecular pore, structural data indicate that the Y4 domain connects the lower and upper parts of the cytosolic crown (9). Specifically, the base of the crown interacts with the prongs exclusively through direct Y4 contacts with the Ubl2-PL2pro segment of the N-terminal part of nsp3 (Fig. 1A and B), thereby stabilizing the overall crown architecture. The first βααβ repeat of Y4 interacts with PL2pro (helix αE) through the outer of the second α-helix H42 (Fig. 7A and B). Within the interface, only two residues G1871 and I1874 are highly conserved within betacoronaviruses (Fig. 7A). Similarly, in other coronaviruses, a small residue (Gly, Ala, or Ser) is consistently found at the first position and a long hydrophobic residue at the second (Fig. S4), creating a complementary surface that permits helix-helix packing between H42 of Y4 and αE of PL2pro. The identification of βααβ structural elements in arteriviruses and tobaniviruses, similar to those found in coronavirus Y4 subdomain, is consistent with the possibility that these viruses may also form DMV pores with a broadly similar organization.

### TM2 domain and Zn-binding motifs

Anchoring the cytosolic crown into the highly curved membrane wall of the DMV pore is a necessary step for molecular pore complex assembly. We therefore hypothesized that the wedge-like topology of the SARS-CoV-2 TM2 domain is conserved across other nidoviruses that contain Y-like regions. To test this, we modeled the TM2-Y regions of representative tobaniviruses and arteriviruses using AlphaFold. Modeling allowed us to align the TM2 sequences of 11 tobaniviruses and 15 arteriviruses despite very low sequence identity (Fig. S9A). Across all modeled species, TM2 adopts a helical wedge-like topology. However, the detailed architecture and spatial arrangement of TM2, Zn-binding motifs, and the adjacent Y domain relative to one another differ between the viral families (Fig. S9B).

To further explore these differences and assess membrane-induced effects on TM2–Y regions, we performed MD simulations of representative viruses from both groups of viruses and compared the results with those obtained for coronaviruses. For subsequent analyses, we selected XTLV and SHFV as representatives of the *Tobaniviridae* and *Arteriviridae* families, respectively, based on the high pLDDT confidence scores of their AlphaFold-predicted models. Each TM2–Y model was generated in the presence of Zn(II) ions and subjected to a 500 ns MD simulation in an all-atom lipid bilayer.

The XTLV TM2 forms a helical hairpin resembling the wedge-shaped topology of SARS-CoV-2 TM2 (Fig. 4B). Although the length of the two hairpin helices varies among different species, all contain a conserved proline at the turn that accentuates the bend and stabilizes membrane insertion (Fig. S9A). The short linker (4–6 unstructured residues) connecting the first two helices to the C-terminal transmembrane α-helix lacks the amphipathic luminal anchor observed in coronaviruses; however, the overall wedge-like orientation of TM2 is preserved.

As in coronaviruses, tobaniviruses have two Zn-binding motifs in the Y1 subdomain. However, in this group, Zn-BM1 is located 2–3 residues downstream of TM2 and does not directly link TM2 to the Y1 domain. Consistent with this positioning, the XTLV Zn-BM1 is less deeply embedded in the membrane (Fig. 4D, middle), while the positively charged surface of Y1 still contacts the bilayer, as observed in coronaviruses. Both Zn(II)-BMs were predicted to adopt octahedral coordination geometry in XTLV. Two water molecules were consistently present within the first hydration shell, and four occupied the second throughout the entire MD simulation. Unlike SARS-CoV-2, however, the water molecules did not penetrate into the hydrophobic core of the lipid bilayer from the cytosolic side of TM2 (Fig. 4B).

On the luminal side, the two helices of XTLV TM2 helical hairpin differ substantially in length—H1 has 15 residues and H2 has 6—producing an asymmetric V-shape with a wider luminal face. Both helices are partially embedded in the membrane and displace lipids of the luminal leaflet, opening a pathway for water to enter the membrane from the luminal side (Fig. 4B).

The TM2 architecture of SHFV differs from that observed in corona- and tobaniviruses. In particular, the first α-helix is unfolded, and the hairpin kink is stabilized by two proline residues (Fig. S9C). Several charged and polar residues on the luminal side are oriented toward the lipid headgroups, suggesting that they play a potential role in local bilayer destabilization. Consistent with this hypothesis, MD simulations revealed transient penetration of water molecules from the luminal side reaching up to the tip of the hairpin region (Fig. 4C).

Unlike coronaviruses and tobaniviruses, arteriviruses possess only one Zn-binding motif, which combines both structural and membrane-interacting functions within the Y1-like domain (Fig. S8). In SHFV, this Zn-binding site is positioned toward the membrane and embedded within the lipid headgroup layer to a degree comparable to that observed for the XTLV (Fig. 4D, bottom).

Taken together, despite structural differences, both tobanivirus and arterivirus TM2 regions adopt a helical wedge topology compatible with membrane embedding and local bilayer perturbation, differing mainly in the specific hairpin architecture. These observations suggest that the wedge-like TM2 arrangement is a conserved feature across nidoviruses replicating in DMVs, potentially facilitating membrane curvature and remodeling during DMV formation.

## DISCUSSION

Our study reveals a remarkable degree of structural conservation in the C-terminal portion of nsp3 across diverse nidoviruses, despite substantial sequence divergence. Using AlphaFold modeling and structure-informed multiple sequence alignments, we demonstrate that the Y1/Y2 region, previously well characterized only in coronaviruses, is topologically preserved in members of the *Tobaniviridae*, *Olifoviridae*, *Gresnaviridae,* and *Arteriviridae* families. We identified a minimal Y1/Y2 fold topology, centered around a Zn-binding Y1 domain and its adjacent Y2 domain, that is retained in all examined vertebrate-infecting nidoviruses, except for a few aquatic species. This conservation implies a common evolutionary origin and suggests an important role for Y1/Y2 in the viral life cycle.

Formation of replicative organelles within host cells is a hallmark of all nidoviruses, and export of newly synthesized viral genomic RNA from these compartments requires a membrane-spanning pore. The cryo-EM structure of the SARS-CoV-2 DMV pore (9) demonstrated that the nsp3 Y region constitutes the lower base of the cytosolic crown of the pore, with hexameric Y1/Y2 domains forming the inner pore channel. The positioning of Y1/Y2 oligomers on the cytoplasmic side of the pore, directly adjacent to the membrane, suggests that these domains may participate in vRNA binding and facilitate its export from the DMVs. Consistent with this notion, a recent study reported that the SARS-CoV-2 Y region, specifically Y1–Y3 subdomains, interacts with the 5′ UTR of the viral genome (39). Our results extend this model, showing that the Y1/Y2 tandems from both tobani- and arteriviruses also form hexameric assemblies *in silico*, utilizing the same conserved structural features, a long α-helix from Y2 and a β-hairpin from Y1, that define the oligomerization interface. This conservation supports the view that pore-forming Y1/Y2 architecture and, potentially, RNA-export mechanisms may represent an evolutionary developed feature of the nidoviruses that infect vertebrates.

Y1 is the most conserved subdomain of Y that was identified by bioinformaticians based on two 100% conserved Zn-binding motifs in tobani- and coronaviruses (19). On the other hand, despite high structural conservation, the Y2 domain shows considerable sequence variability. The topology conservation with high sequence variability may be driven by selection on fold stability rather than specific functional residues needed to mediate intermolecular interactions. One reason the Y2 domain remained unrecognized in arteriviruses and tobaniviruses is its compact size relative to coronavirus Y2, coupled with low sequence similarity. Coronavirus Y2 domains are approximately 1.5 times larger due to an insert of an additional antiparallel β-sheet (Fig. 5B). This β-sheet is not involved in inter-subunit contacts within the assembled DMV pore and is positioned on the exterior surface of the crown. Its peripheral location and absence in other nidoviruses imply a coronavirus-specific functional adaptation. Supporting this conjecture, several residues within this β-sheet are highly conserved across coronaviruses and surface-exposed. For example, in SARS-CoV-2, nsp3 Y1695 is invariant in betacoronaviruses (Fig. S3) and is replaced by phenylalanine in alphacoronaviruses. This aromatic residue is surrounded by conserved positively charged residues, forming a basic patch that could potentially mediate interactions with nucleic acids. It remains unclear whether this potential RNA-binding site participates in the reported interaction with the 5′ UTR of the viral genome (39) or serves another function unrelated to vRNA export.

The C-terminal part of the nsp3 Y region, comprising the Y3 and Y4 subdomains in coronaviruses, is absent in other nidoviruses. Among these, Y3 is the least conserved, whereas Y4 is the second most conserved subdomain after Y1. In the DMV pore, Y4 plays an important role connecting the lower base of the crown with the upper part through the direct interaction with the Ubl2-PL2pro region of nsp3N. However, the interaction interface does not exhibit high conservation, suggesting that the interaction may depend more on the overall protein structure than on specific side-chain contacts. The presence of similar structural elements in tobaniviruses and arteriviruses suggests that these viruses may also form DMV pores with a comparable overall architecture.

While the oligomeric Y1/Y2 tandem forms the cytosolic base of the pore crown, functional integration into the membrane depends on the upstream TM2 segment. Despite extremely low sequence conservation (Fig. S1A), TM2 domains across all coronaviruses adopt the same helical wedge-like topology: a wide luminal region formed by a helical hairpin embedded in the membrane, followed by amphipathic helices, and ending with a long membrane-spanning α-helix (Fig. 2A and D). As shown by Huang et al., the inner and outer membranes of the DMV are fused around the pore, resembling the architecture of the nuclear pore complex (9). TM2 anchors the pore crown to a highly curved membrane with a circular cross-section — wider on the luminal side and narrower at the pore channel. The wedge-like shape of TM2 perfectly accommodates this geometry: the wide luminal portion allows the insertion of six TM2 domains with bulky amphipathic helices, while the narrow cytosolic end positions only the C-terminal portion of α-helix H4 to point toward the pore channel. The flexible angles between membrane-inserted helices (H1, H2, and H4) and amphipathic helices (H2’ and H3) also enhance adaptation to varying membrane curvature. We observed a similar wedge-like topology in tobaniviruses and arteriviruses, although TM2 in these viruses lacks the amphipathic luminal anchor present in coronaviruses. This structural conservation suggests that TM2 acts as a mechanical adaptor coupling the rigid cytosolic crown to the varying curvature of the lipid bilayer.

Another shared feature among these viral families is the presence of 100% conserved Zn(II)-binding motifs near the membrane surface. Both tobaniviruses and coronaviruses possess two Zn-BMs: one at the TM2–Y1 junction and another within Y1 that stabilizes its fold. Arteriviruses contain a single Zn-BM that fulfills both roles. Our simulations show that all Zn-binding motifs adopt an unusual octahedral coordination geometry in which two water molecules remain tightly bound to the Zn(II) ion and four additional waters form a second hydration shell. In all cases, the membrane-interacting Zn-binding motifs are embedded in the lipid headgroup layer (Fig. 4D). In SARS-CoV-2, Zn-BM1 involves a residue from TM2 that pulls the hydrated Zn(II) ion deeper into the bilayer, opening a pathway for water penetration into the hydrophobic core. Our MD simulations further demonstrate that water molecules permeate deeply into the bilayer, suggesting that Zn-BM1 may locally destabilize the membrane.

Previous studies have shown that Zn(II) can induce membrane bending by bridging phosphate groups of neighboring lipids and promoting headgroups dehydration (40, 41). Furthermore, it has been demonstrated that when Zn(II) interacts with only one leaflet of a phospholipid bilayer, Zn(II) binding decreases the area per lipid in the exposed leaflet, creating tension that drives membrane curling (41). Dehydration also increases surface hydrophobicity, facilitating membrane fusion (40). Nsp3 TM2-Y anchors the crown on the side of the pore where the inner and outer DMV membranes fuse. In our MD simulations, Zn(II) was consistently coordinated by two water molecules along with the side chains of four protein residues. However, the proximity of phosphate groups suggests that one or both water molecules could, in principle, be replaced by phosphate oxygens from adjacent lipids. Although the precise timing of inner and outer membrane fusion during DMV formation is unknown, it is reasonable to propose that pore assembly and fusion occur concurrently or in close succession. We speculate that during Y1/Y2 hexamer formation, Zn-BM1 likely releases Zn(II), which, once free, may further promote local membrane dehydration and facilitate fusion. This mechanism is consistent with the MD simulations, but further experimental studies are needed to confirm it.

Overall, we propose that Zn-BM1 acts during the initial stages of DMV biogenesis, promoting local membrane destabilization and curvature that precede membrane fusion necessary for pore formation. Upon pore assembly, Zn(II) release allows disengagement of the Y1 subdomain from the membrane for subsequent oligomerization, linking early membrane remodeling with pore formation. The persistence of this motif in tobani- and arteriviruses, its unusual coordination geometry, and its consistent membrane embedding across viral families underscore its conserved role in the evolution of nidovirus replication organelles.

In the monomeric state, SARS-CoV-2 nsp3 Y adopts a compact architecture in which all Y subdomains are tightly packed. In contrast, within the assembled DMV pore, the Y region is extended, and the close Y2-Y3 interaction is lost (9) (Fig. 1D). The compact form was first observed in the X-ray structure of the SARS-CoV-2 CoV-Y (Y2–Y4) domain (23), and our previous NMR studies detected no flexible linker between Y2 and Y3 subdomains, consistent with a stable, compact configuration in solution (42). In the MD simulations, SARS-CoV-2 nsp3 Y remained compact during the entire 500 ns without signs of opening. Although AlphaFold predictions tend to favor compact conformations, this agreement across methods suggests that the compact form of alpha- and betacoronavirus Y domains is biologically relevant, possibly representing a pre-assembly or membrane-associated state. In contrast, gamma- and deltacoronaviruses are predicted to adopt more extended Y conformations, potentially reflecting functional divergence beyond DMV pore formation.

Our comparative modeling across all known nidoviruses reveals that the nsp3 TM2-Y region is far more conserved than previously appreciated. Structural analyses identify homologous TM2-Y1/Y2 modules in four additional nidoviral families *— Tobaniviridae*, *Gresnaviridae*, *Olifoviridae*, and *Arteriviridae* — extending far beyond *Coronaviridae*. Despite extensive sequence divergence, all retain a wedge-like TM2 topology suited for insertion into highly curved membranes and a Y1/Y2 tandem capable of oligomerization. These parallels point to a unified structural mechanism underlying DMV pore formation across vertebrate-infecting nidoviruses.

### Conclusion

The C-terminal TM2-Y region of nsp3 represents a conserved structural module that is essential for membrane remodeling and pore formation across multiple nidoviral families. Despite extreme sequence divergence, our structural and computational analyses suggest a common architecture in which the Y1/Y2 tandem forms an oligomerization scaffold for the cytosolic crown of the DMV pore, while a wedge-shaped TM2 anchors the crown within curved membranes.

The discovery of homologous Y regions in members of the *Tobaniviridae* and *Arteriviridae* families suggests that the molecular pore architecture observed in SARS-CoV-2 might not be unique to coronaviruses but instead might represent a shared and ancient feature of nidovirus replication. The strict conservation of the Zn-binding motif at the TM2-Y1 junction, despite its loss of Zn(II) coordination in the assembled pore, points to a critical function of this region in previous stages of DMV formation. Our MD simulations suggest that membrane-interacting Zn-BM1 induces localized membrane destabilization, creating conditions for membrane bending, fusion, and subsequent pore assembly during DMV biogenesis.

Together, these findings suggest a unified structural mechanism for nidovirus DMV pore formation, in which conserved transmembrane and cytosolic elements cooperate to remodel host membranes and enable vRNA export. This work offers a preliminary framework for investigating replication organelles biogenesis in coronaviruses and other nidoviruses that pose threats to human health as well as the meat and fish industries and identifies the C-terminal region of nsp3 as a promising target for broad-spectrum antiviral development.

## MATERIALS AND METHODS

### AlphaFold model generation

Initially, all structures were calculated using AlphaFold 2 (22) hosted on NMRbox platform (43) equipped with A100 and H100 GPUs. Protein sequences were retrieved in FASTA format from the UniProt database, following their annotations. For proteins lacking domain annotation, overlapping sequence fragments of various lengths were submitted for calculation until the TM2-Y region could be identified or ruled out. The full list of analyzed viruses, including their current taxonomy according to the International Committee on Taxonomy of Viruses (ICTV) and UniProt IDs, is provided in Table S1.

The final models were calculated using the AF3 server (24) with the appropriate number of Zn(II) ions included. Model quality assessment utilized per-residue confidence metrics provided by AlphaFold, including predicted Local Distance Difference Test (pLDDT) for monomeric structure and, in addition, predicted template modeling (pTM) and interface predicted alignment (ipTM) for oligomers. pLDDT can range from 0 to 100. A pLDDT of 90–100 indicates high prediction accuracy; 70–90 signifies a confident backbone conformation; 50–70 implies low confidence in local structure prediction, and values below 50 suggest poor prediction or an unstructured region. pTM reflects the overall accuracy of the protein complex structure predicted by AlphaFold-Multimer and is expected to exceed 0.5. ipTM is a measure of how accurate the relative positioning of different subunits is, with 0.8–1.0 indicating high reliability. The presence of linkers or disordered regions can reduce iPTM scores even when the overall complex was predicted correctly. Models were ranked based on mean pLDDT or pTM and visualized using the PyMOL Molecular Graphics System, Version 2.5.2 (Schrödinger, LLC). Electrostatic surface potentials for Y domain oligomers were computed using the Adaptive Poisson-Boltzmann Solver (APBS) plugin in PyMOL (44).

### Structure-curated multiple sequence alignments

Initial multiple sequence alignments (MSAs) for coronaviruses were executed using ClustalW (45). Each group — 20 alphacoronaviruses, 15 betacoronaviruses, 5 gammacoronaviruses, and 7 deltacoronaviruses — was aligned independently at first. Following this, we modeled structures for selected representatives from each group. The alignments were manually refined based on secondary structure elements observed in these models. In cases of ambiguity, the 3D structural alignments of the corresponding structures were examined in PyMOL to guide the correction. Similarly, MSAs for tobani- and arteriviruses were initially performed with MUSCLE (46) and then manually adjusted using the AlphaFold predicted structural models. Per-residue identity scores were calculated from the curated MSAs using in-house Python scripts.

### MD simulations

All-atom MD simulations were performed using GROMACS (47) on the NMRbox platform (43) using L40S GPUs. The top-ranked AF3 models for TM2-Y regions from SARS-CoV-2 nsp3 (residues 1499–1945, UniProt P0DTC1), Xinzhou toro-like virus (XTLV) nsp3 (residues 3534–3850, UniProt A0A1L3KIY4), and Simian Hemorrhagic Fever Virus (SHFV) nsp2 (residues 992–1219, UniProt Q68772) were used as starting points.

The assembly of an explicit lipid bilayer was performed with the CHARMM-GUI server (48–50) with a lipid ratio of 9:1 for POPC:POPG for both membrane leaflets. Initial protein orientation in the lipid bilayer was determined using the PPM 2.0 server (51). The system was solvated with TIP3P water models (52) and sodium chloride for protein charge compensation. The CHARMM36m force field (53) was applied with periodic boundary conditions in all directions.

The initial energy minimization was performed using the steepest descent method until convergence. The system was equilibrated over six stages (for 1.875 ns in total) with gradually reduced positional restraints. The temperature was maintained at 303.15 K using a velocity rescaling thermostat with a coupling time of 1 ps (54), and pressure was maintained at 1 bar using exponential relaxation pressure coupling with a time constant of 5 ps. All hydrogen bonds were constrained using the LINCS algorithm (55), and Zn(II) ions were constrained with harmonic restraints to maintain the experimentally observed coordination distances (56).

Production simulations were performed with the Nose-Hoover thermostat (57). The electrostatic forces were calculated using the Particle-Mesh Ewald algorithm (58) with a cutoff of 1.2 nm. Van der Waals potential was calculated with a 1.2 nm cutoff with a smoothing to zero function applied after 1.0 nm.

The trajectories were analyzed using standard GROMACS utilities.

## Data Availability

The AF3 models of hexamers formed by XTLV and SHFV Y-like domains (Fig. 6) are available in ModelArchive (www.modelarchive.org) with the accession codes ma-1ap7f and ma-v9dsy, respectively. The final structures of TM2-Y regions in full-atom lipid bilayers after 500 ns MD simulation for SARS-CoV-2, XTLV, and SHFV (Fig. 4) are available on Zenodo at https://doi.org/10.5281/zenodo.17653358.

## SUPPLEMENTAL MATERIAL

Supplemental material jvi.02038-25-s0001.pdf Table S1; Fig. S1 to S9.

ASM does not own the copyrights to Supplemental Material that may be linked to, or accessed through, an article. The authors have granted ASM a non-exclusive, world-wide license to publish the Supplemental Material files. Please contact the corresponding author directly for reuse.
