# Supplementary material for: The DMV pore-forming TM2–Y region of SARS-CoV-2 nsp3 exhibits structural conservation beyond the coronavirus family
Source: J Virol. 2026 Apr 3;100(5):e02038-25. doi: 10.1128/jvi.02038-25 (PMC13185587; doi:10.1128/jvi.02038-25)
Supplement: Supplemental material — Table S1; Fig. S1 to S9. [file jvi.02038-25-s0001.pdf]

The DMV pore-forming TM2–Y region of SARS-CoV-2 nsp3 exhibits structural conservation beyond the coronavirus family

Alexandra Pozhidaeva, Jeffrey C. Hoch, Yulia Pustovalova[#]

Supplemental Information

Table of Contents

TABLE S1. FULL LIST OF VIRUSES USED FOR BIOINFORMATIC ANALYSIS. 2

FIGURE S1. SEQUENCE ALIGNMENT AND STRUCTURAL COMPARISON OF NSP3 TM2 REGIONS ACROSS ALL CURRENTLY KNOWN CORONAVIRUSES. 6

FIGURE S2. SEQUENCE ALIGNMENT OF NSP3 Y1 SUBDOMAIN ACROSS ALL CURRENTLY KNOWN CORONAVIRUSES. 8

FIGURE S3. SEQUENCE ALIGNMENT OF NSP3 Y2 SUBDOMAIN ACROSS ALL CURRENTLY KNOWN CORONAVIRUSES. 9

FIGURE S4. SEQUENCE ALIGNMENT OF NSP3 Y4 SUBDOMAIN ACROSS ALL CURRENTLY KNOWN CORONAVIRUSES. 10

FIGURE S5. COMPARISON OF HEXAMERS FORMED BY α AND γ CORONAVIRUS NSP3 Y REGIONS. 12

FIGURE S6. MOLECULAR DYNAMICS ANALYSIS OF THE SARS-COV-2 NSP3 Y REGION. 13

FIGURE S7. SEQUENCE ALIGNMENT OF NSP3 Y-LIKE REGIONS FROM TOBANIVIRUSES. 15

FIGURE S8. SEQUENCE ALIGNMENT OF NSP2 Y-LIKE REGIONS FROM ARTERIVIRUSES. 17

FIGURE S9. SEQUENCE ALIGNMENT OF TM2 REGIONS FROM TOBANI AND ARTERIVIRUSES. 18

REFERENCES 19

Table S1. Full list of viruses used for bioinformatic analysis.

Taxon[1]			UniProt/NCBI	Name	Acronym	Taxon ID	Y[2] (Y/N)
Suborder	Family	Genius					
Cornidovirineae	Coronaviridae	Alpha-coronavirus	A0A240FW11	Suncus murinus coronavirus X74	SMCoV-X74	2848125	Y
			A0A096XNJ3	Lucheng Rn rat coronavirus	LRNV	1508224	Y
			A0A7D5BTQ3	Mink coronavirus 1	MCoV	1913642	Y
			A0A0K2BMC4	Porcine transmissible gastroenteritis coronavirus	TGEV	693997	Y
			A8JNZ0	Rhinolophus bat coronavirus HKU2	Rh-BatCoV-HKU2	693998	Y
			P0C6U6	Human coronavirus NL63	HCoV-NL63	277944	Y
			A0A1L2KGB4	NL63-related bat coronavirus	BtCoV-NL63r	1920748	Y
			P0C6U2	Human coronavirus 229E	HCoV-229E	11137	Y
			B1PHK0	Miniopterus bat coronavirus HKU8	Mi-BatCoV-HKU8	694001	Y
			B1PHJ3	Miniopterus bat coronavirus 1	Mi-BatCoV-1A	694000	Y
			P0C6W0	Scotophilus Bat coronavirus 512	Sc-BatCoV-512	693999	Y
			P0C6Y4	Porcine epidemic diarrhea virus	PEDV	229032	Y
			S5Z1D1	Bat coronavirus CDPHE15	BtCoV-CDPHE15	1384461	Y
			A0A0U1UZC3	Myotis ricketti alphacoronavirus Sax-2011	BtMr-SAX2011	1503289	Y
			A0A0U1UZ30	Rhinolophus ferrumequinum alphacoronavirus HuB-2013	BtRf-HuB2013	1503292	Y
			K4JZB7	Hipposideros Bat coronavirus HKU10	Hi-BatCoV-HKU10	1241932	Y
			A0A6B9KDG7	Hipposideros pomona bat coronavirus CHB25	Hi-BatCoV-CHB25	2691598	Y
			A0A0U1WHG1	Nyctalus velutinus alphacoronavirus SC-2013	BtNv-SC2013	1503291	Y

			A0A3G8EYU2	Pipistrellus kuhlii coronavirus 3398	Pk-BatCoV-3398	2492658	Y
			A0A4Y5QNN6	Tylonycteris bat coronavirus HKU33	Tr-BatCoV-HKU33	2586420	Y
		Beta-coronavirus	P0DTC1	Human Severe acute respiratory syndrome coronavirus 2	SARS-CoV-2	2697049	Y
			P0C6U8	Human Severe acute respiratory syndrome coronavirus	SARS-CoV	694009	Y
			A0A088DIE1	Bat Hp-betacoronavirus Zhejiang 2013	BtHp-Zhejiang	1541205	Y
			A0A2Z4EVM4	Eidon helvum bat coronavirus C704	Ei-BatCoV-C704	2849735	Y
			A0A1B3Q5W8	Rousettus bat coronavirus GCCDC1	RoBat-GCCDC1	1892416	Y
			P0C6W5	Rosettus Bat coronavirus HKU9	Ro-BatCoV-HKU9	694006	Y
			U5KNA9	Hedgehog coronavirus 1	EriCoV	1385427	Y
			P0C6W3	Tylonycteris Bat coronavirus HKU4	Ty-BtCoV-HKU4	694007	Y
			R9UQ29	Middle East respiratory syndrome-related coronavirus	MERS-CoV	1335626	Y
			P0C6W4	Pipistrellus Bat coronavirus HKU5	Pi-BtCoV-HKU5	694008	Y
			P0C6X2	Human coronavirus HKU1	HCoV-HKU1	443239	Y
			Q66WN6	Murine hepatitis virus	MHV	11138	Y
			A0A2H4MYY9	Myodes coronavirus 2JL14	MrufCoV-2JL14	2847997	Y
			P0C6U7	Human coronavirus OC43	HCoV-OC43	31631	Y
			A0A0A7UXR0	China Rattus coronavirus HKU24	ChRCoV-HKU24	1590370	Y
		Gamma-coronavirus	B2BW31	Beluga whale coronavirus SW1	BWCoV	694015	Y
			A0A4D6FWB1	Canada goose coronavirus CB17	CGCoV-CB17	2569586	Y

			A0A0F6WGL5	Duck coronavirus 2714	DCoV-2714	2849730	Y
			P0C6Y1	Avian infectious bronchitis virus	aIBV	11122	Y
			A0A0K1RN38	Chicken infectious bronchitis virus	chIBV	2847808	Y
		Delta-coronavirus	H9BR16	Night heron coronavirus HKU19	NHCoV-HKU19	1159904	Y
			H9BR24	Wigeon coronavirus HKU20	WiCoV-HKU20	1159908	Y
			H9BR34	Common moorhen coronavirus HKU21	CMCoV-HKU21	1159902	Y
			H9BQX7	Porcine coronavirus HKU15	PorCoV-HKU15	1159905	Y
			B6VDY6	Munia coronavirus HKU13	MuCoV-HKU13	572289	Y
			H9BQZ1	White-eye coronavirus HKU16	WECoV-HKU16	1159907	Y
			B6VDV9	Bulbul coronavirus HKU11	BuCoV-HKU11	572288	Y
Tornidovirineae	Tobaniviridae	Torovirus	P0C6V7	Berne Virus	BEV	11156	Y
			A0A097P9K6	Porcine torovirus	PoTV	237020	Y
		Bafinivirus	Q008X5	White bream virus	WBV	766180	Y
		Pregotovirus	A0A076PY83	Ball python nidovirus 1	BPNV1	1986118	Y
		Bostovirus	A0A0F6PMZ2	Bovine nidovirus TCH5	BNV TCH5	1631554	Y
		Sectovirus	A0A1L3KJ67	Xinzhou nematode virus 6	XzNV6	1923774	Y
		Oncotshavirus	A0A0E3GHV1	Chinook salmon bafinivirus	CSBV	1611837	Y
		Infratovirus	A0A1L3KIY4	Xinzhou toro-like virus	XTLV	1923777	Y
		Lyctovirus	A0A888WKT1	Veiled chameleon serpentovirus A	VCSV-A	2806429	Y
		Vebetovirus	A0AAE7P737	Veiled chameleon serpentovirus B	VCSV-B	2806430	Y
		Septovirus	A0AAE6TXC3	Septovirus foka	SVF	3071292	Y
Arnidovirineae	Arteriviridae	Alphaarterivirus	P19811	Equine arteritis virus	EAV	299386	Y
		Betaarterivirus	Q04561	Porcine reproductive and respiratory syndrome virus 1	PRRSV1	11049	Y

		Gammaarterivirus	Q83017	Lactate dehydrogenase elevating virus	LDV	300016	Y
		Deltaarterivirus	Q68772	Simian hemorrhagic fever virus	SHFV	38143	Y
		Epsilonarterivirus	A0A0F6PT34	Simian hemorrhagic encephalitis virus	SHEV	1965068	Y
		Zetaarterivirus	A0A1L5YNJ4	Kibale red colobus virus 1	KRCV1	1885929	Y
		Etaarterivirus	X2D5C6	Kibale red colobus virus 2	KRCV2	1936072	Y
		Thetaarterivirus	A0A089FY51	Mikumi yellow baboon virus 1	MYBV-1	1546177	Y
		Iotaarterivirus	A0A0B6C113	DeBrazza's monkey arterivirus	DeMAV	1965063	Y
		Kappaarterivirus	A0A6M3Q8N5	Wobbly possum disease virus	WPDV	1118369	Y
		Lambdaarterivirus	A0A0B5JQL5	African pouched rat arterivirus	APRAV	1965064	Y
		Muarterivirus	A0A1Z2RX77	Olivier's shrew virus 1	OSV1	2012619	Y
		Nuarterivirus	A0A2H4MXL2	RtClan arterivirus	RtClanAV	2847271	Y
	Gresnaviridae	Cyclophivirus	A0A2P1GMV4	Guangdong greater green snake arterivirus	GGGSAV	2116442	Y
	Olifoviridae	Kukrinivirus	A0A2P1GMT8	Hainan oligodon formosanus arterivirus	HOFAV	2116440	Y
	Cremegaviridae	Pontunivirus	A0A2P1GMW5	Chinese broad-headed pond turtle arterivirus	CBHPTAV	2116345	N
		Sicregavirus	YP_010799370 (NCBI)	Trionyx sinensis hemorrhagic syndrome virus	TSHSV	1705352	N
Ronidovirineae	Roniviridae	Okavirus	B3U1H4	Yellow head virus	YHV	96029	N
	Euroniviridae	Charybnivirus	A0A1L3KIX8	Beihai Nido-like virus 2	BNLV2	1922351	N
Nanidovirineae	Nanghoshaviridae	Chimshavirus	A0A2P1GMU6	Nanhai ghost shark arterivirus	NGSAV	2116441	N
	Nanhypoviridae	Sajorinivirus	A0A2P1GMW1	Wuhan japanese halfbeak arterivirus	WJHAV	2116443	N
Abnidovirineae	Abyssoviridae	Alphaabyssovirus	YP_009553215.1 (NCBI)	Aplysia abyssovirus 1	AAbV	2283236	N
Monidovirineae	Mononiviridae	Alphamononivirus	A0A386JUR1	Planarian secretory cell nidovirus	PSCNV	2100419	N
Mesnidovirineae	Mesoniviridae	Alphamesonivirus	A0A679DYN4	Cavally virus	CavV	1041929	N
	Medioniviridae	Turrinivirus	A0A1L3KIW1	Beihai Nido-like virus 1	BNlV1	1922350	N

[1] According to International Committee on Taxonomy of Viruses (ICTV) website.

[2] Indicates whether a domain similar to the SARS-CoV-2 nsp3 Y region was identified in this species.

Figure S1. Sequence alignment and structural comparison of nsp3 TM2 regions across all currently known coronaviruses.

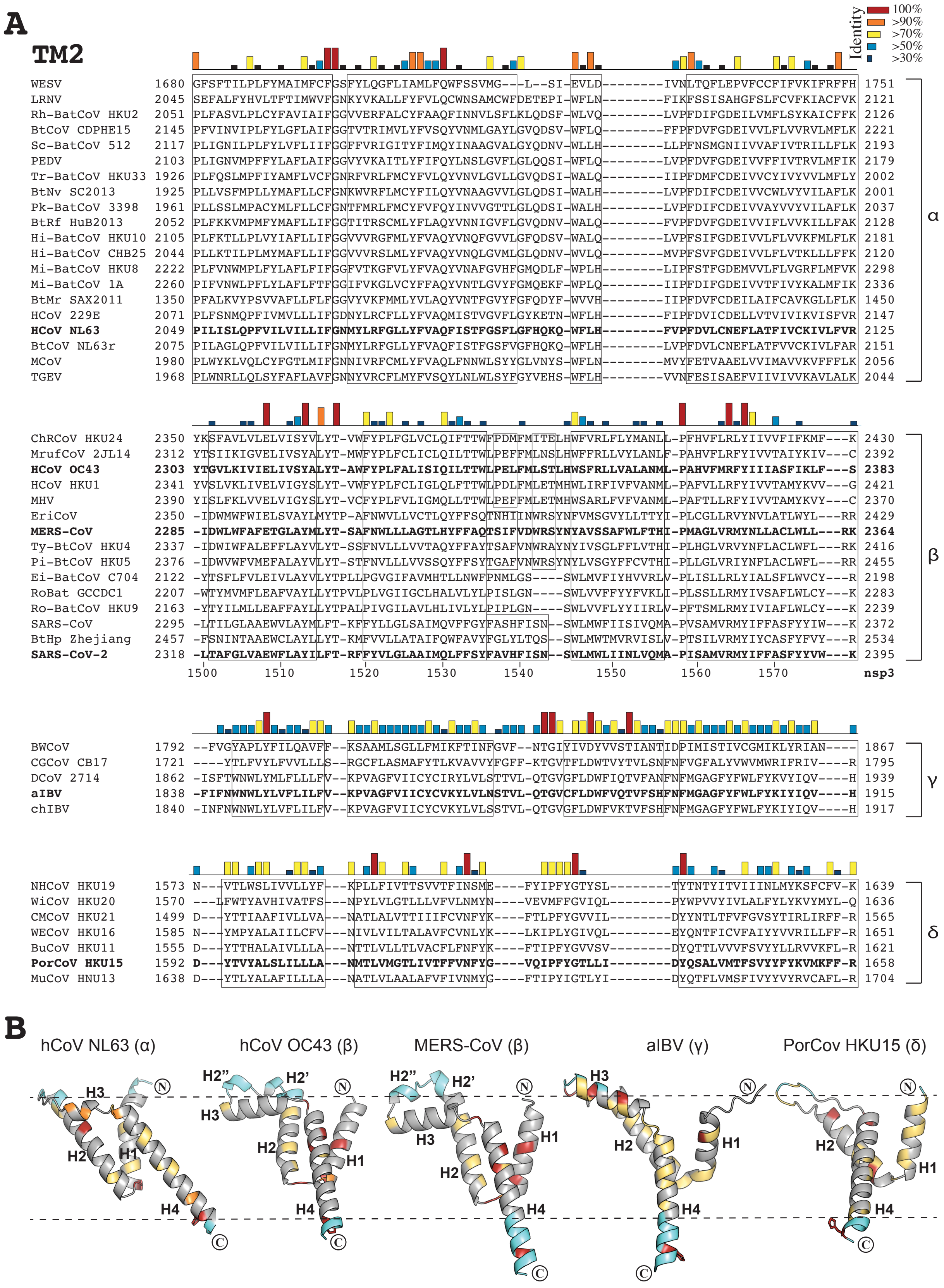

Figure S1. Sequence alignment and structural comparison of nsp3 TM2 regions across all currently known coronaviruses.

(A) MSA of TM2 regions from all currently known coronaviruses (see Table S1). The first and last residues corresponding to nsp3 TM2 region are indicated for each species using pp1a numbering; the nsp3 numbering is shown for SARS-CoV-2 only. Sequences were first aligned within each subfamily, followed by cross-family alignment The DMV pore-forming TM2–Y region of SARS-CoV-2 nsp3 exhibits structural conservation beyond the coronavirus family. Helical segments of the TM2 regions are indicated by boxes. Sequence identity scores within each subfamily were calculated after the final structure-based alignment using an in-house Python script.

(B) Cartoon representations of AlphaFold 2 (1) models of nsp3 TM2 from selected coronaviruses. Dashed lines indicate the membrane boundaries predicted by the PPM 3.0 server (2). Structures are colored according to residue conservation (identity scores > 70%); water-accessible regions of TM2 are highlighted in cyan.

Figure S2. Sequence alignment of nsp3 Y1 subdomain across all currently known coronaviruses.

Y1

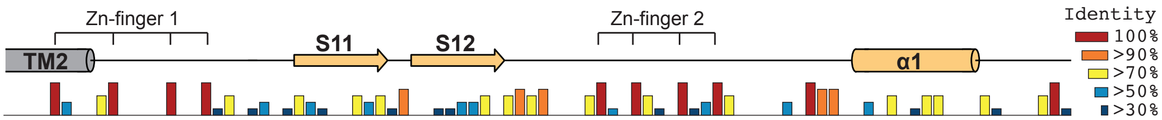

α–CoVs

```
SMCoV-X74      1751 ---HHVVFGCTKSFCKYCSKAAKQKKFLVSTIVNGSSKSFYVNANGGGK-FCNKHNFYCKGCESYGVGNTFINESVADYLSDYVKHRVVATA 1838
LRNV           2121 ---KHVVFGCNDVKCLSCSRSAKYDRISCDTIVNGVKRSFYVKANGG-KSFCEKHQFFCQNCDSYGVGCTFINDHVAKEVANVTRTAVRATG 2208
Rh-BatCoV-HKU2 2126 ---KHVVFGCDKPSCVACCKSAKLKRIAMDTIVNGSRRSFYVNANGGAK-LCKKHNFFCVACDSYGSGHTYINDHIANELHNVTKLHVKPTG 2213
BtCoV-CDPHE15  2221 ---KHVLFGCDKPSCLACSKSARLKRVPVSTIVSGANKSFYVNANGGT-SFCKKHNFYCVNCDSYGLGCTFINDVVAAEVGNVTKLNVQATG 2308
Sc-BatCoV-512  2193 ---KHVLFGCDKPSCIACSKSAKLTRVPLQTILQGVTKSFYVNANGG-KKFCKKHNFFCVDCDSYGYGCTFINDVIAPELSNVTKLNVIPTG 2280
PEDV           2179 ---KHVCLGCDKASCVACSKSARLKRVPVQTIFQGTSKSFYVHANGGSK-FCKKHNFFCLNCDSYGPGCTFINDVIATEVGNVVKLNVQPTG 2266
Tr-BatCoV-HKU33 2002 ---YHVCFGCEKPNCVACSKSARLTRIPVQTIVNGSTKVVYVTANGGRRAFCKKHNFFCVNCDSYGVGCTFINEHVSRELSNVTKTNVQPTG 2090
BtNv-SC2013    2001 ---KHVCFGCEKPSCVACSKSARLTRIPIQTVVNGSTKVVYVAANGGNRPFCKKHNFFCVNCDSYGVGCTFINEHVARELANVTKTNVQPTG 2089
Pk-BatCoV-3398 2037 ---KHIFFGCEKPNCIACSKSCRMTRIAVQTIVNGSSKMVYVTASGGNKPFCKKHNFFCVNCDSYGTGNTFINEHVARELSNVVKTSVQPTG 2125
BtRf-HuB2013   2128 ---KHIVFGCDKPSCVACTKSARLTRVPMQTIVNGSNKSFYVAANGGKK-FCKKHNFFCVNCDSYGPGNTFINDVVAAEISNVVKTSVAPTA 2215
Hi-BatCoV-HKU10 2120 ---KHVFLGCERPSCVACSKSARLTRIPMQTIVNGSNRSFYVVANGGKK-FCEKHKFFCVNCDSYGQGNTFINDVVAAEVSNVVKTNVIATG 2268
Hi-BatCoV-CHB25 2298 ---KHVLLGCDKPSCVACSKSARLTRVPMQTIVNGSNRSFYVVANGGK-SFCSKHKFFCVNCDSYGPGCTFINDFVAIELSNVVKTNVIPTS 2207
Mi-BatCoV-HKU8 2298 ---KHVCFGCDKASCVACSKSARLTRVPMQTIVCGANKSFYVTANGGK-NFCSRHNFFCVNCDSHGPGNTFINEVVARELSNVVKSSVKPTD 2385
Mi-BatCoV-1A   2336 ---KHVCFGCEKPSCVACSKSARLNRVPMQTIVNGANKLFYVVANGGS-SYCHKHKFFCLNCDSYGPGNTFINETVARELSNVVKTNVQPTG 2423
BtMr-SAX2011   1450 ---KHVIMGCDSPSCIACSKSARLRRVPMTTVVNGANKSFYVTANGGTK-FCTKHNFFCVDCGSYGNNSTFINDHVARELSNVVKTSVRPTG 1537
HCoV-229E      2147 ---RHVLFGCENPDCIACSKSARLKRFPVNTIVNGVQRSFYVNANGGSK-FCKKHRFFCVDCDSYGYGSTFITPEVSRELGNITKTNVQPTG 2234
HCoV-NL63      2125 ---RHIIVGCNNADCVACSKSARLKRVPLQTIINGMHKSFYVNANGGT-CFCNKHNFFCVNCDSFGPGNTFINGDIARELGNVVKTAVQPTA 2212
BtCoV-NL63r    2151 ---RHIIVGCNDADCIACSKSARLKRVPLQTIVNGMNKSFYVNANGGTH-FCNKHNFFCVNCDSFGPGNTFINGDIARELGNVVKTTVQPTA 2238
MCoV           2056 ---KHYWYGCDKADCKSCSKIAKQNRIPVNVVVNGSIKSVYVHTNGGSK-FCKRHNFYCKNCDSYGSDNTFICHDIVKDLSSAVKHSVYATD 2143
TGEV           2044 ---KHIVFSCSNPSCKTCSKTARQTRISISVVVNGSMKTVYVPGHGTGK-LCKKHNFYCKNCDSYGFENTFICDEIVRDLSNSVKQTVYATD 2131
```

β–CoVs

```
ChRCoV-HKU24   2430 KLFRHVVHGCNKPGCLFCYKRNKSVRVKCSTVVGGMLRYYDVMANGGI-NFCNKHQWNCIGCDSYKPGNTFITVEAAAELSKEFKRTINPTD 2520
MrufCoV-2JL14  2392 CLFRHIVHGCCKPGCLFCYKRNRSVRVKCSTIVGGMLRYYDVMANGGT-GFCNKHQWNCIGCDSYKPGNTFITVEAAAELSKEFRRPVHATD 2482
HCoV-OC43      2383 SLFKHVAYGCSKSGCLFCYKRNRSLRVKCSTIVGGMIRYYDVMANGGT-GFCSKHQWNCIDCDSYKPGNTFITVEAALDLSKELKRPIQPTD 2473
HCoV-HKU1      2421 GFIRHIVYGCNKAGCLFCYKRNCSVRVKCSTIVGGVIRYYDITANGGT-GFCVKHQWNCFNCHSFKPGNTFITVEAAIELSKELKRPVNPTD 2511
MHV            2370 CLCRHVMYGCSKPGCLFCYKRNRSVRVKCSTVVGGSLRYYDVMANGGT-GFCTKHQWNCLNCNSWKPGNTFITHEAAADLSKELKRPVNPTD 2460
EriCoV         2429 RFYNHVINGCKDTACMLCYKRNRLTRVEASTVVCGVKRTFYITANGGI-SFCSRHNWNCVDCDTTGLGHTFICEEVANDLTTSLRRLVKPTD 2519
MERS-CoV       2364 KFYQHVINGCKDTACLLCYKRNRLTRVEASTVVCGGKRTFYITANGGI-SFCRRHNWNCVDCDTAGVGNTFICEEVANDLTTALRRPINATD 2454
Ty-BtCoV-HKU4  2416 KFYSHVINGCKDTACLLCYKRNRLTRVEASTIVCGTKRTFYIAANGGT-SYCCKHNWNCVECDTAGVGNTFICTEVANDLTTTLRRLIKPTD 2506
Pi-BtCoV-HKU5  2455 RFYNHVINGCKDTACLLCYKRNRLTRVEASTVVCGSKRTFYIVANGGT-SFCCRHNWNCVDCDTAGIGNTFICEEVANDLTTSLRRLVKPTD 2545
Ei-BatCoV-C704 2198 RGLCHVRFGCNNVSCLMCYKRNGATRVECSTIVAGVKRSFYVNANGGT-YFCTKHNWNCVNCDSYSVDSTFISRPVALDLSAQFKRPINHTD 2288
RoBat-GCCDC1   2283 KGFVHVRYGCTNVSCLMCYKRNIATRVECSTVVNGVKRVFYVNANGGT-YYCTKHNWNCVNCDTYTVDSTFISRPVALDLSAQFKRPINHTD 2373
Ro-BatCoV-HKU9 2239 KGFLHVRYGCNNVACLMCYKKNVAKRIECSTVVNGVKRMFYVNANGGTH-FCTKHNWNCVSCDTYTVDSTFICRQVALDLSAQFKRPIIHTD 2329
SARS-CoV       2372 KSYVHIMDGCTSSTCMMCYKRNRATRVECTTIVNGMKRSFYVYANGGR-GFCKTHNWNCLNCDTFCTGSTFISDEVARDLSLQFKRPINPTD 2462
BtHp-Zhejiang  2534 RAYVHVVSGCQDTRCLMCYKRNLARRVECHTVVQGSRKSFYVHANGGT-GFCEKHAWNCINCDSYGPGNTFICDEVAVDLSTTFKRPVKPTD 2624
SARS-CoV-2     2395 KSYVHVVDGCNSSTCMMCYKRNRATRVECTTIVNGVRRSFYVYANGGK-GFCKLHNWNCVNCDTFCAGSTFISDEVARDLSLQFKRPINPTD 2485
                       1580      1590      1600      1610      1620       1630      1640      1650      1660      nsp3
```

γ–CoVs

```
BWCoV          1864 RIANHYFYGCKNPACDKCHAATVSERTELSCVVGSKRFPYYVYANSVAK-MCGKHMWCCKNCDVFGEGNTFISTAIVGDLTDKIRRKVKATS 1954
CGCoV-CB17     1792 RIVRHVFY-CKDVNCVYCGKLAKNARYEVTLVVAGKKHSVYVYTNAGY-FYCKQHNWYCRNCNKFGHQNTFISPEVAGDLSDKLKRRIMPTS 1881
DCoV-2714      1937 IQVHHIMY-CKDITCEVCKRVARSNRHEVSVVVNGRKQLVHVYTNSGY-SFCKRHNWYCRNCDKYGHQNTFMSPEVAGELSEKLKRHVKPTA 2026
aIBV           1913 IQVHHILY-CKDVTCEVCKRVARSNRQEVSVVVGGRKQIVHVYTNSGY-NFCKRHNWYCRNCDDYGHQNTFMSPEVAGELSEKLKRHVKPTA 2002
chIBV          1639 VQVHHILY-CKDGTCGVCKRVARSTRQEVSVVVGGRQQIVHVYTNSGY-NFCKRHNWYCRNCDDYGHQNTFMSPEVAGELSEKLKRHVKPTA 2004
```

δ–CoVs

```
NHCoV-HKU19    1639 ---KHLVSGCSKTGCSICAKKSVYTVLNVETVIQGRKFVSQVSTNGGTK-FCEKHNFYCLNCDNPMDDGTFIPVEAVESLSKATNTHVKPTS 1726
WiCoV-HKU20    1636 ---QHISVGCKKPTCKLCIKKNIAKRVTVEVVLQGRKHYTMVNTNGGT-NFCDKHDFFCENCEKGGVDGTYIPIEVIESLSKATGLAPKPTG 1723
CMCoV-HKU21    1565 ---RHVSKGCKSPTCTICSRIRTSPTITVETIVQGRKYPSTIITNEGS-VVCKQHNFYCKNCDAI-NPDTFIPVEAVESLAKVTKLDVKSTA 1651
WECoV-HKU16    1651 ---RHLAYGCKKPNCNLCAKMKIAPTLTVDTVVQGRKFPSVIHTNGGVK-ICPNHNFYCKDCDST-NPTTFIPTEAVESLSKITRLHVKPTA 1737
BuCoV-HKU11    1620 ---RHIAKGCKTPTCTICSKLRIPPTITVDTIVQGRKYPSVVQTNAGFK-ICKEHNFYCKNCSQQ-NPDTFIPTEAIESLSRATRLNVKSTA 1706
PorCoV-HKU15   1658 ---RHLTHGCKIPTCVVCAKLRTPPTITVETVVQGRKYPSVIETNGGF-TICKEHNFYCKDCSLQ-TPGTFIPTEAIESLSRATRLSVKPTA 1744
MuCoV-HNU13    1704 ---RHLAKGCKQPTCTLCSKMRTSPTITVETIVQGRKYPSVVETNGGF-SICKEHNFYCKNCTAE-KPGTFIPTEAVESLSRTTKLSVKPTA 1790
```

Figure S2. Sequence alignment of nsp3 Y1 subdomains across all currently known coronaviruses. The full list of all analyzed species is in Table S1. The first and last residues corresponding to nsp3 Y1 region are indicated for each species using pp1a numbering; the nsp3 numbering is shown for SARS-CoV-2 only.

The top panel shows the secondary structure elements of SARS-CoV-2 nsp3 Y1 and identity scores calculated across all analyzed species after MSA was manually adjusted using AlphaFold structural models.

Figure S3. Sequence alignment of nsp3 Y2 subdomain across all currently known coronaviruses.

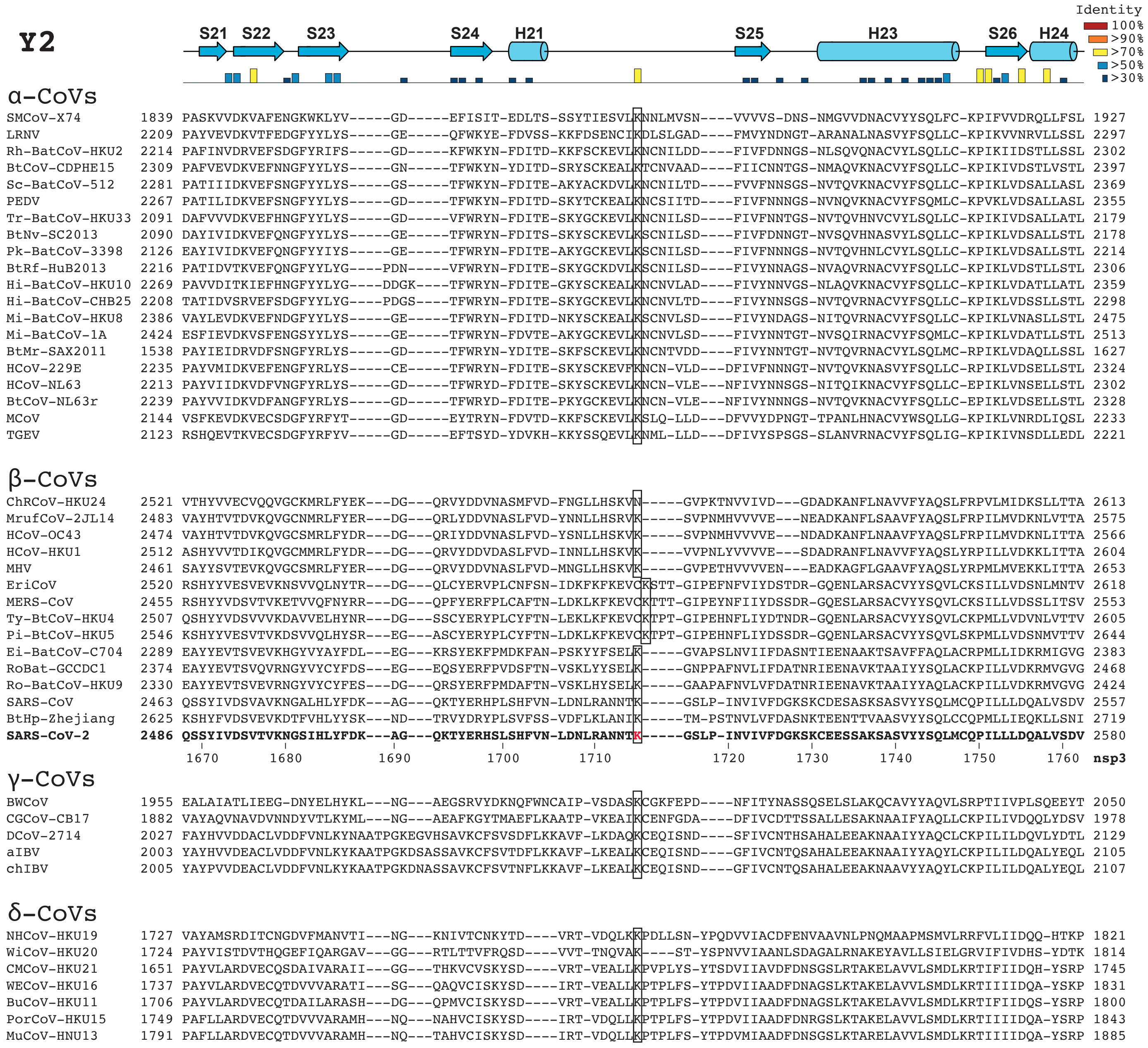

Figure S3. Sequence alignment of nsp3 Y2 subdomains across all currently known coronaviruses. The full list of all analyzed species is in Table S1. The first and last residues corresponding to nsp3 Y2 region

are indicated for each species using pp1a numbering; the nsp3 numbering is shown for SARS-CoV-2 only. The top panel shows the secondary structure elements of SARS-CoV-2 nsp3 Y2 and identity scores calculated across all analyzed species after MSA was manually adjusted using AlphaFold structural models.

Figure S4. Sequence alignment of nsp3 Y4 subdomain across all currently known coronaviruses.

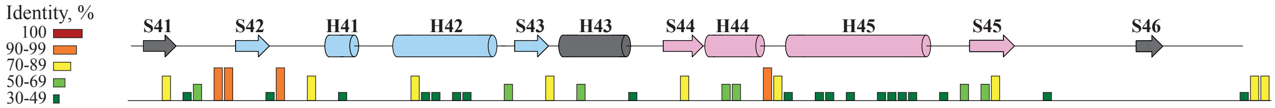


```
α-CoVs

SMCoV-X74        1985 VLLTDRSYNNFTITYA-KIGDKLS-KTDQAVCLRANVGIINHNVAVKENISVVWYYKNFLQLSEEGKKYIVKTTKAKGVGFYITFNDIEANTQAPVTRFQ------KNAG 2086
LRNV             2253 ILLCNDSANNFVTTYA-KAGTRIG-TADLAVFNRESVREVSHAVLARNKTTIVWSVNVFSKLSGDTQDYIVRTTKAKGLLFLLTFNDHVNTQNLPCTMVN-----SKSAG 2355
Rh-BatCoV HKU2  2361 VLITDTSFNNFCTSYA-KPAEKLS-SFDLAHCMRSGAKVVNHNVLIKEKMPIVWNCVDFAKLSVDARKYIVKTAKVKGVTFLLTVNNNVMETTLPCVSVL-----QKQGA 2463
BtCoV CDPHE15   2457 LLLTDISFNNFITSYA-KPEEKMS-VHDLAICMRSGARVANHNVLVKENVPVVWSAEAFNALSEESRKYVVKTSKVKGLTFMLSFNNVRMHTTIPSVAIS-----GKKGG 2559
Sc-BatCoV 512   2429 VLISDVSFNNLIVSYA-KPEEKLA-VHDIANCMRVGAKVVNHNVLTKDNVPVVWLAKDFIALSEEARKYIVRTTKTKGINFMLTFNDRRMHLTIPTISVA-----NKKGA 2531
PEDV             2415 VLLTDMSFNNFTTSYA-KPEEKFP-VHDIATCMRVGAKIVNHNVLVKDSIPVVWLVRDFIALSEETRKYIIRTTKVKGITFMLTFNDCRMHTTIPTVCIA-----NKKGA 2517
Tr-BatCoV HKU33 2238 ILLSDLSFNNVVTTYA-KPEEKMS-THDMAVCLRTGAKIVNHNVLTKENIPVVWLARDFQSLSEDCRKYVVKTTKQKGVTFFITFNDTRMNLSIPVVSFS-----CKKGG 2340
BtNv SC2013      2237 ILLTDISFNNFITTYA-KPEEKFA-THDAAVCMRSGSKIVNHNVLIKENAAVVWYQRDFHALSEDCRKYIVKTSKQKGVNIMITFNDTRMNVAIPAVNFV-----CKKGG 2339
Pk-BatCoV 3398  2273 VLMTDTSFNNLITTYA-KPEEKLA-THDAAVCMRSGAKVVNHNVIVKENMPIVWSARDFHALSEDCRKYIVKTTKQKGINFMLSLNETRMHTTVPAVNFV-----CKKGG 2375
BtRf HuB2013     2364 VLLTDLSFNNFVTSYA-KPEEKLS-THDIAACMRGGSKVVNHNVLVKENVPIVWLAKDFNMLSEEGRKYVVKTAKIKGINFMLTFNENRMQTTIPTVSIA-----GKKGS 2466
Hi-BatCoV HKU10 2417 TLLSDLSYNNFVTSYA-KPEEKLS-THDLAACMRGGAKVVNHNILVKENVPIVWYAKDFNMLSEEGRRYVVKTAKLKGVNFLLTFNDNRMQTQIPVVSVT-----TKCGS 2519
Hi-BatCoV CHB25 2357 VLITDLSYNNFLTTYA-KPEEKLA-THDLASCMRSGAKVVNHNILVKENTPIVWFAKDFNMLSDEGRKYVVKTAKLKGVNFMLSFNDNRMQTSIPIVSVT-----TKCGS 2459
Mi-BatCoV HKU8  2533 VLLSDTSCNNLVTSYA-KPEEKLS-VHDLGTCMRFGAKVVNHNVLIKENVPVVWLASEFHLLSEEGRKYIVKTTKAKGVTFLLTFNSNPMQLNIPSTSIV-----NKRAA 2635
Mi-BatCoV 1A    2571 VLLSDNSFNNLLTSYA-KPEEQLS-THDVATCMRFNAKVVNHNVLIKENVPIVWLARDFQQLSEEGRKYLVKTTKAKGVTFLLTFNTNAMNVKLPAISIV-----NKKGA 2673
BtMr SAX2011     2291 VLLSEVSFNNFITSYA-KPEEKLS-AFDCAQCVRAGAKIVNHNVIVKEKLSIVWLAKDFQALSAEGRKYIVKTSKVKGLNFILTLNDNAAQCSVPVVSMM-----SKKGA 2393
HCoV 229E        2382 VLLSDLSFNNFVSSYA-KPEEKLS-AYDLACCMRAGAKVVNANVLTKDQTPIVWHAKDFNSLSAEGRKYIVKTSKAKGLTFLLTINENQAVTQIPATSIV-----AKQGA 2484
HCoV NL63        2360 VLLSDLSFNNFFISYA-KPEDKLS-VYDIACCMRAGSKVVNHNVLIKESIPIVWGVKDFNTLSQEGKKYLVKTTKAKGLTFLLTFNDNQAITQVPATSIV-----AKQGA 2462
BtCoV NL63r     2386 ILLSDLSFNNFFTSYA-KPEEKLS-AYDVACCMRAGSKVVNHNVLTKESTPVVWSAKDFNTLSQEGKKYLVKTTKAKGLSFLLTFNDNQAITQVPATSIV-----SKQGA 2488
MCoV             2291 VLMTDLSFNNFWLTYA-KPEDNLT-SFDIANCIRANAKAINHSVLSKENVPIVWSATDFVKLSNEAKHLLIKTAKAKGVTIMITLNTNVMSHNLPAVAIG------RKSG 2392
TGEV             2279 ILITDRSFNNFWPSKV-KPGSSGVSAMDIGKCMTSDAKIVNAKVLTQRGKSVIWLSQDFAALSSTAQKVLVKTFVEEGVNFSLTFNAVGSDDDLPYERFTE--SVPPKSG 2385

β-CoVs

ChRCoV HKU24     2697 LDMTDESCNNLVPTYI-KS-DNIV-AADLGVLIQNNCKHVQGNVAKMAGVSCIWHVDVYNQLSSDFQHKLKKACVKTGLKLKLTYNKQAANVSIL-TMPF-----SLKGG 2797
MrufCoV 2JL14    2660 IEITDDSYNNLVPTYI-KT-DTIS-AADLGVLIQHNCKHVQGNVAKMASVACVWSFDAYNQLSSDFQHKLKKACCKTGLKLKVTYNKQFANVSIL-TTPF-----SLKGG 2760
HCoV OC43        2650 LELTDESCNNLVPTYL-KS-DNIV-AADLGVLIQNSAKHVQGNVAKIAGVSCIWSVDAFNQFSSDFQHKLKKACCKTGLKLKLTYNKQMANVSVL-TTPF-----SLKGG 2750
HCoV HKU1        2738 LEFTDENYNNLVPTYL-KS-DNIV-AADLGVLIQNGAKHVQGNVAKAANISCIWFIDAFNQLTADLQHKLKKACVKTGLKLKLTFNKQEASVPIL-TTPF-----SLKGG 2838
MHV              2737 VDFTDESCNNLVPTYV-KS-DTIV-AADLGVLIQNNAKHVQANVAKAANVACIWSVDAFNQLSADLQHRLRKACSKTGLKIKLTYNKQEANVPIL-TTPF-----SLKGG 2837
EriCoV           2702 IELVTDSFNNYIPSYV-KP-DSIA-TVDLGCLIDLKAASVNPASMRNANGACVWNIDAYLKLSDSLKRQIRVACRKCSLNFKLTTSKLRAQDNIL-SVKF--SVTKFVGG 2805
MERS-CoV         2637 IQITNESYNNYVPSYV-KP-DSVS-TSDLGSLIDCNAASVNQIVLRNSNGACIWNAAAYMKLSDALKRQIRIACRKCNLAFRLTTSKLRANDNIL-SVRF--TANKIVGG 2740
Ty-BtCoV HKU4    2682 IQLTTECYNNYVPGYI-KP-DSIN-TLDLGCLIDLKAASVNQTSMRNANGACVWNSGDYMKLSDSFKRQIRIACRKCNIPFRLTTSKLRAADNIL-SVKF--SATKIVGG 2785
Pi-BtCoV HKU5    2728 LQLTTEGFNNYVPSYI-KP-DSVA-TADLGCLIDLNAASVNQTSIRNANGACIWNSSDYMKLSDSLKRQIRIACRKCNIPFRLTTSRLRSADNIL-SVKF--SATKLSGG 2831
Ei-BatCoV C704  2469 WDWSVGPYNNLIPTYL-KQ-DTLT-TLEVGQLISCSARYVNANIAKSSAINIVWRFSDFIKLSESLRRQIRIAARKTGLNLQLTTSSLKADVTCV-VTPI-----RVVGG 2569
RoBat GCCDC1     2554 WEWTTASWNNLVPTYL-KQ-ETLS-TLEVGQLITSNARYINANMAKSAAVQIVWRYADFARLSESVRRQLKIAARKTGLSLQLTTSNLKADVACV-VTPF-----KVVGG 2654
Ro-BatCoV HKU9  2510 WEWTTDSWNNLVPTYI-KQ-DTLS-TLEVGQFMTANAKYVNANIAKGAAVNLIWRYADFIKLSESMRRQLKVAARKTGLNLLVTTSSLKADVPCM-VTPF-----KIIGG 2610
SARS-CoV         2640 LEVTGDSCNNFMLTYN-KV-ENMT-PRDLGACIDCNARHINAQVAKSHNVSLIWNVKDYMSLSEQLRKQIRSAAKKNNIPFRLTCATTRQVVNVI-TTKI-----SLKGG 2740
BtHp Zhejiang    2802 VDITKDSYNDFVLTYN-KV-DSMT-PYDLGVCIEVGARHVNANVAKANAAALIWNIDHYLLLSESFRRQIRSSARKNGLQFKLTCSKNRQVTPFL-TTKI-----MFSGG 2902
SARS-CoV-2       2663 IEVTGDSCNNYMLTYN-KV-ENMT-PRDLGACIDCSARHINAQVAKSHNIALIWNVKDFMSLSEQLRKQIRSAAKKNNLPFKLTCATTRQVVNVV-TTKI-----ALKGG 2763
                          1850      1860        1870      1880      1890      1900      1910      1920      1930      1940          nsp3

γ-CoVs

BWCoV            2122 LTPSQAAYNNAVPNSTCNLDAY....QVKSAFVQFGVKYVDFKMALNDSKIAILQYDFLKSLPGNYLPSLLMRIRQAGVTLRVTTSQIMQQD-------------TLGG 2223
CGCoV CB17       2038 LSFTNDGYTNVVPSYGFDTSKLS--ARDRGFMINAGASIAN--LKIRNAPPVVWRYSDLKNLSETCLKYLFSGVNKSGSKFFVTRADCKQVISCH-TAKLILDKNVKAGG 2142
DCoV 2714        2192 LSYTSDGFTNVVPSYGFDLGKLT--PRDKGFLINADACIAN--LKVRNAPPVVWNYHDLIKLSDNCLKFLVSGVIKSGAKFYVTRSDVKQVITCL-TQKLLLDK--KAGG 2294
aIBV             2164 VDYTGDGFTNVIPSYGIDTGKLT--PRDRGFLINADASIAN--LRVKNAPPVVWKFSELIKLSDSCLKYLISATVKSGVRFFITKSGAKQVIACH-TQKLLVEK--KAGG 2266
chIBV            2166 VEYTGDGFTNVIPSYGIDIDKLT--PRDRGFLINADASIAN--LRVKNAPPVVWKFSELIKLSDSCLKYLISATVKSGVRFFITKSGVKQVIACH-TQKLLVEK--KAGG 2268

δ-CoVs

NHCoV HKU19      1875 LDLTMDNPNNTVPHYAFDFNSL--PSEDKSTLMDFGIGSSV--LKGTSY-NIILPYNLLVKLSVKTIIKLKSAAMKNGVKLLVTPSTRVIRGTLSVIPFNI-----KTGG 1974
WiCoV HKU20      1868 IEFTVDPPNNTIPHYAFDFASI--MPEDQALLKQHECGSGV--MKGTGI-NCVLSANLVKLLAPKTLVKLRNAASRNGIRLCVSPCTRVLRTSIPVVPFG------MKGG 1966
CMCoV HKU21      1799 LDFTIDEPNHVLPHYAFDFSSL--SVEDQSVLLDMGCVKGN--LKGTNV-GVVLTANLVTRLTQQALRVISNAASRNGVTCSVTPSTRVLRGNIVTQPFAR-----IKAG 1898
WECoV HKU16      1885 LDFTIDNPNHILPHYAFDFSTL--SAEDQSVLIETGCAKGN--LKGTNV-GVVLSANVVTRLSNSAIRVIANAASRNGVTCSVTPSTITLRGNIATQPLQR-----LKAG 1984
BuCoV HKU11      1855 LDFTIDNPNHILPHYAFDFNTL--SAEDQSVLLEAGCAKGN--LKGTNV-GVVLAANLVMRLSQSAIRIIANAASRNGVTCAVTPSNITLRGNIATQPFQR-----VKAG 1954
PorCoV HKU15     1897 LDFTIDNPNNILPHYAFDFSTL--NAEDQSTILESGCAKGN--LKGTNV-GVVLSASLVTRLSQQAIRVIANAASRNGVTCAVTPSTLVMRGNIATQPLTR-----IKAG 1997
MuCoV HNU13      1939 LDFTIDNPNNILPHYAFDFTTL--TADDQATIIETGCAKGN--LKGTGV-GVILSANLVTRLSQSALRIIANAASRNGVTCAVTPSTLVMRGNIATQPLTR-----LKAG 2039
```

Figure S4. Sequence alignment of nsp3 Y4 subdomains across all currently known coronaviruses. The full list of all analyzed species is in Table S1. The first and last residues corresponding to nsp3 Y4 region are indicated for each species using pp1a numbering; the nsp3 numbering is shown for SARS-CoV-2 only. The top panel shows the secondary structure elements of SARS-CoV-2 nsp3 Y4 and identity scores calculated across all analyzed species after MSA was manually adjusted using AlphaFold structural models.

Figure S5. Comparison of hexamers formed by α and γ coronavirus nsp3 Y regions.

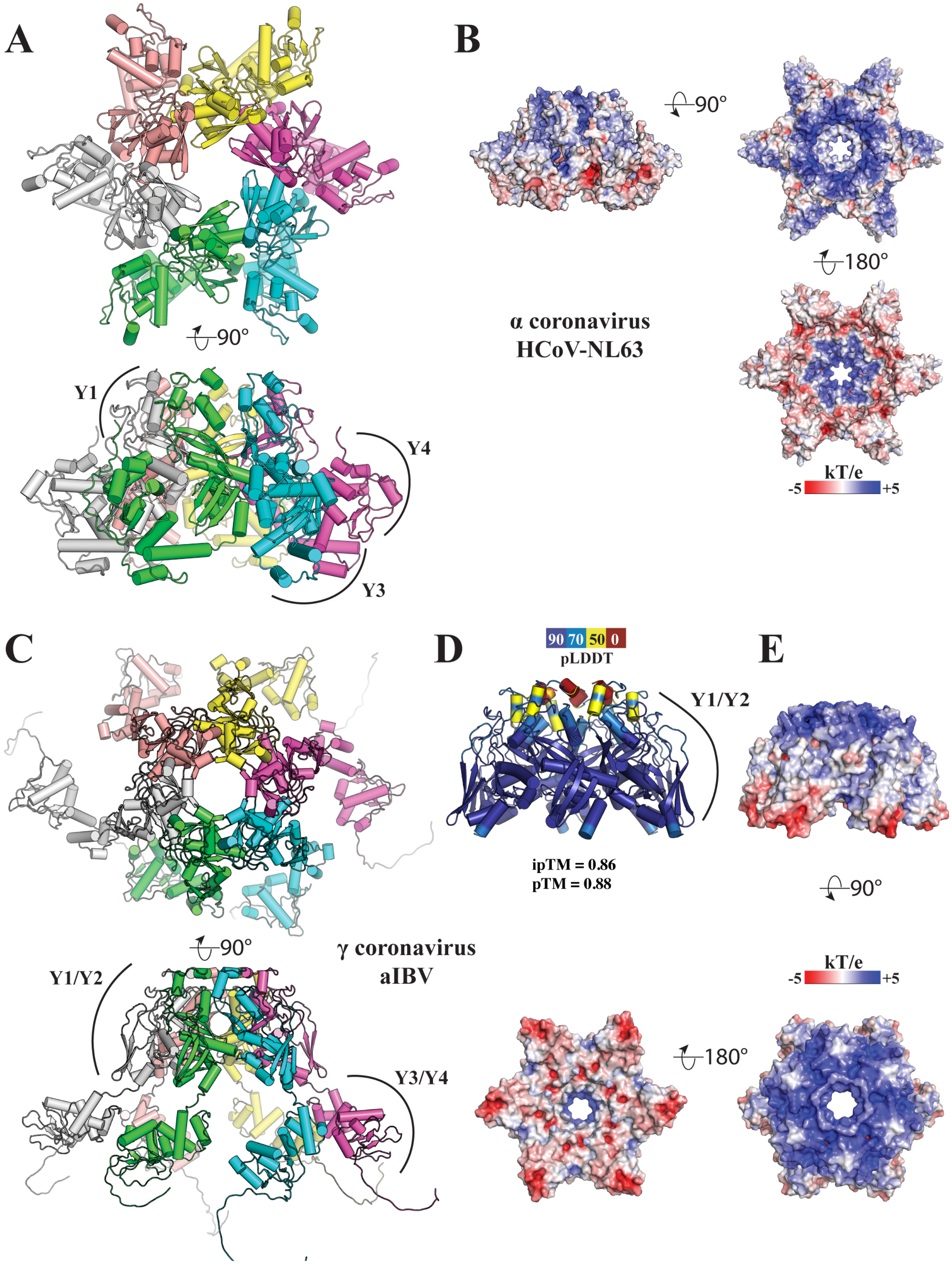

Figure S5. Comparison of hexamers formed by α and γcoronavirus nsp3 Y regions. (A, C) AlphaFold-predicted hexamers of nsp3 Y from α coronavirus HCoV-NL63, which adopts a compact conformation, and γ coronavirus aIBV, which forms an extended conformation. (D) AlphaFold-predicted hexamer of the aIBV nsp3 Y1–Y2 region, colored by pLDDT. ipTM and pTM scores are indicated. (B, E) Electrostatic potential surfaces of the Y1/Y2 hexamers from HCoV-NL63 and aIBV, respectively, calculated using APBS plugin in PyMOL (3).

Figure S6. Molecular dynamics analysis of the SARS-CoV-2 nsp3 Y region.

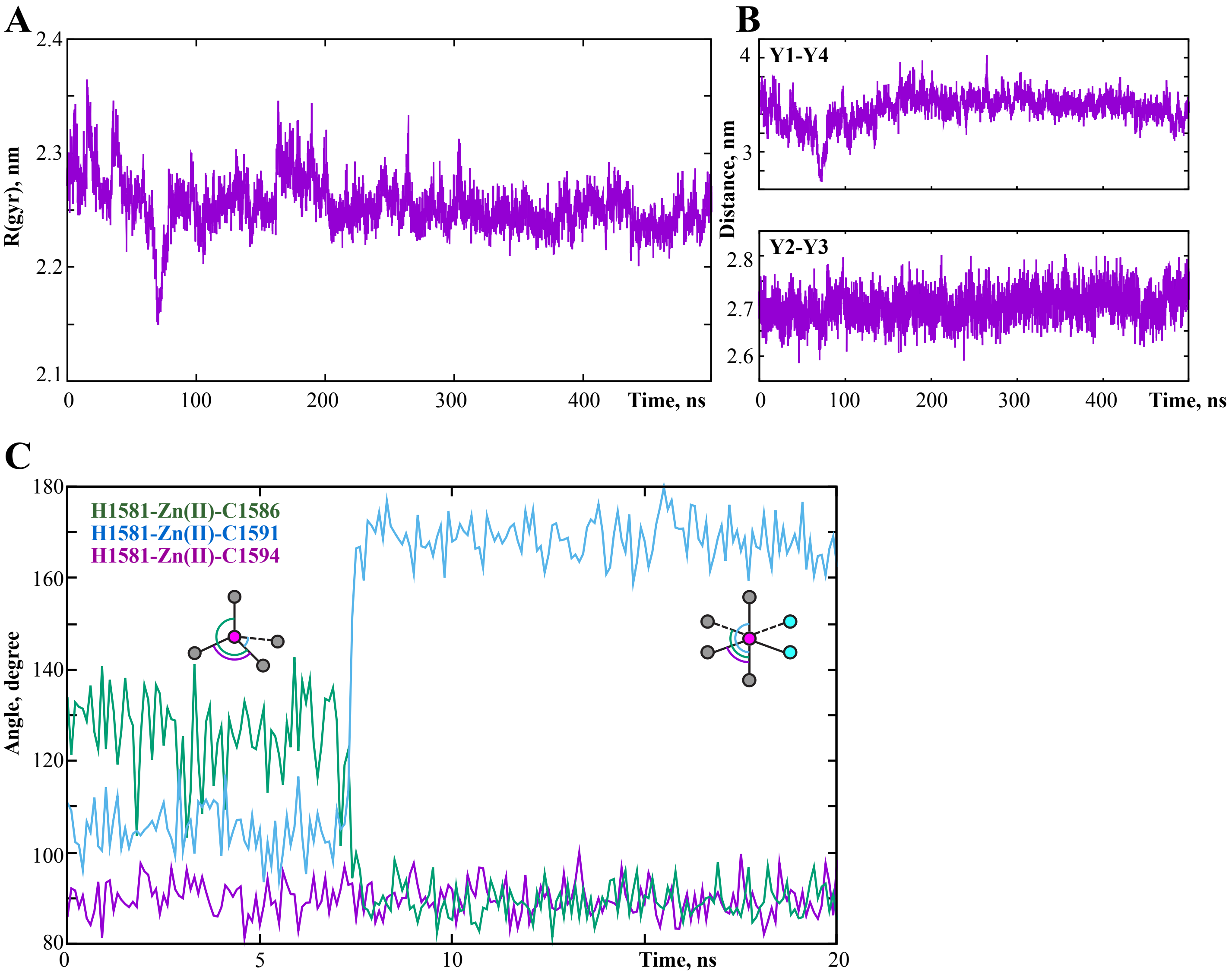

Figure S6. Molecular dynamics analysis of the SARS-CoV-2 nsp3 Y region.

(A) Radius of gyration calculated for the nsp3 Y region (residues 1598-1945).

(B) Distances between domain pairs measured throughout the 500-ns MD simulation: (top) the distance between the center of the Y1 β-hairpin and the centroid of the Y4 domain, and (bottom) the distance between the centroids of Y2 and Y3.

(C) Angles between Zn(II)-coordinating atoms within Zn-BM1 measured during the first 20 ns of the simulation, corresponding to the period when the coordination geometry transition occurs.

Figure S7. Sequence alignment of nsp3 Y-like regions from tobaniviruses.

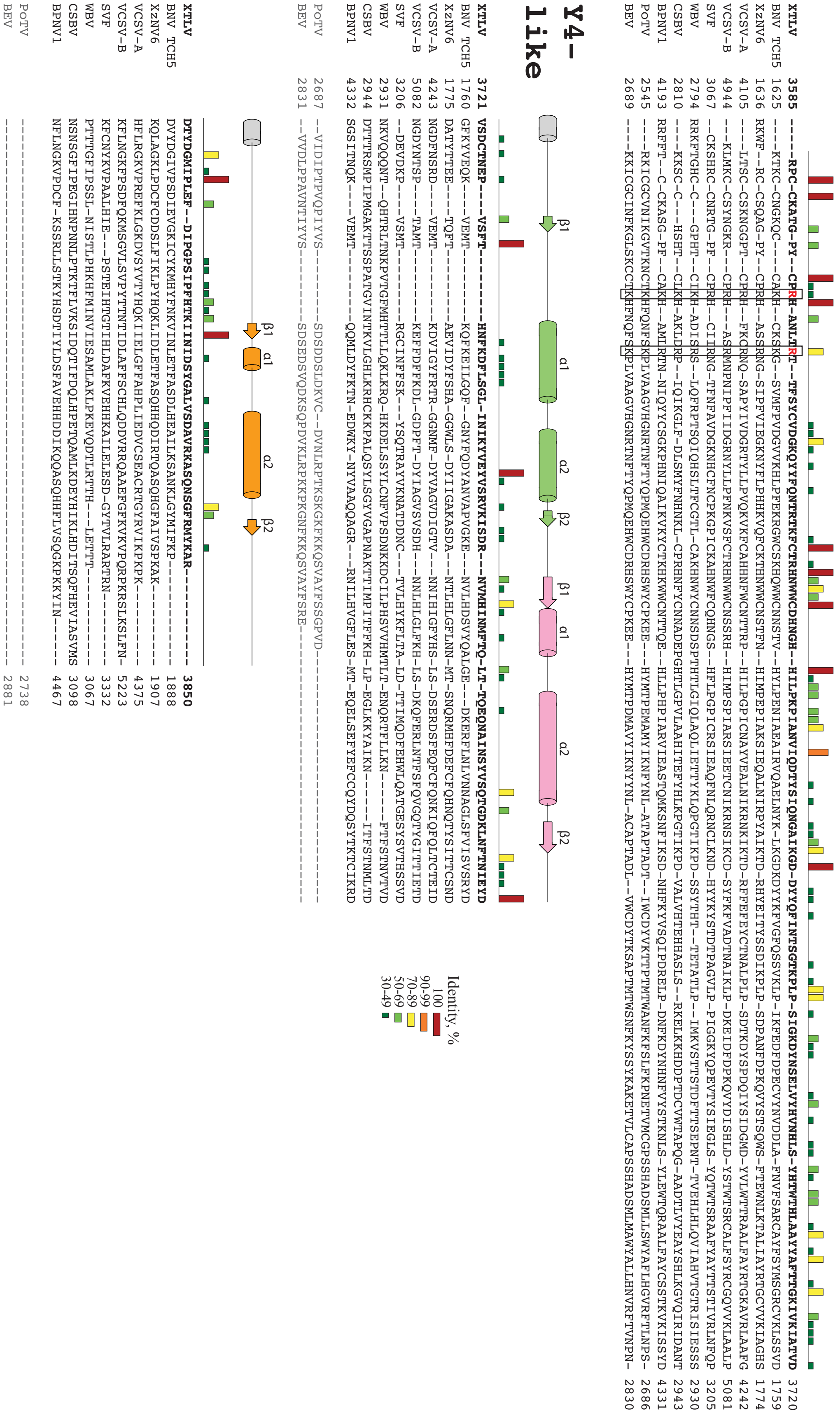

Figure S7. Sequence alignment of nsp3 Y-like regions from tobaniviruses (see Table S1). The first and last residues of analyzed region are indicated for each species using pp1a numbering. Secondary structure elements for XTLV nsp3 are schematically shown at the top. TheY1 and Y2 secondary structure elements are colored in yellow and blue, respectively. The conserved positively charged residues R3597 and R3603 of XTLV are highlighted in red, and their corresponding positions are indicated by boxes in all other tobaniviruses. The structural elements of the βααβ repeats are colored green, pink and orange. The identity scores were calculated across all tested species after MSA was manually adjusted using AlphaFold structural models. PoTV and BEV viruses were excluded from identity scores calculation for Y4-like region.

Figure S8. Sequence alignment of nsp2 Y-like regions from arteriviruses.

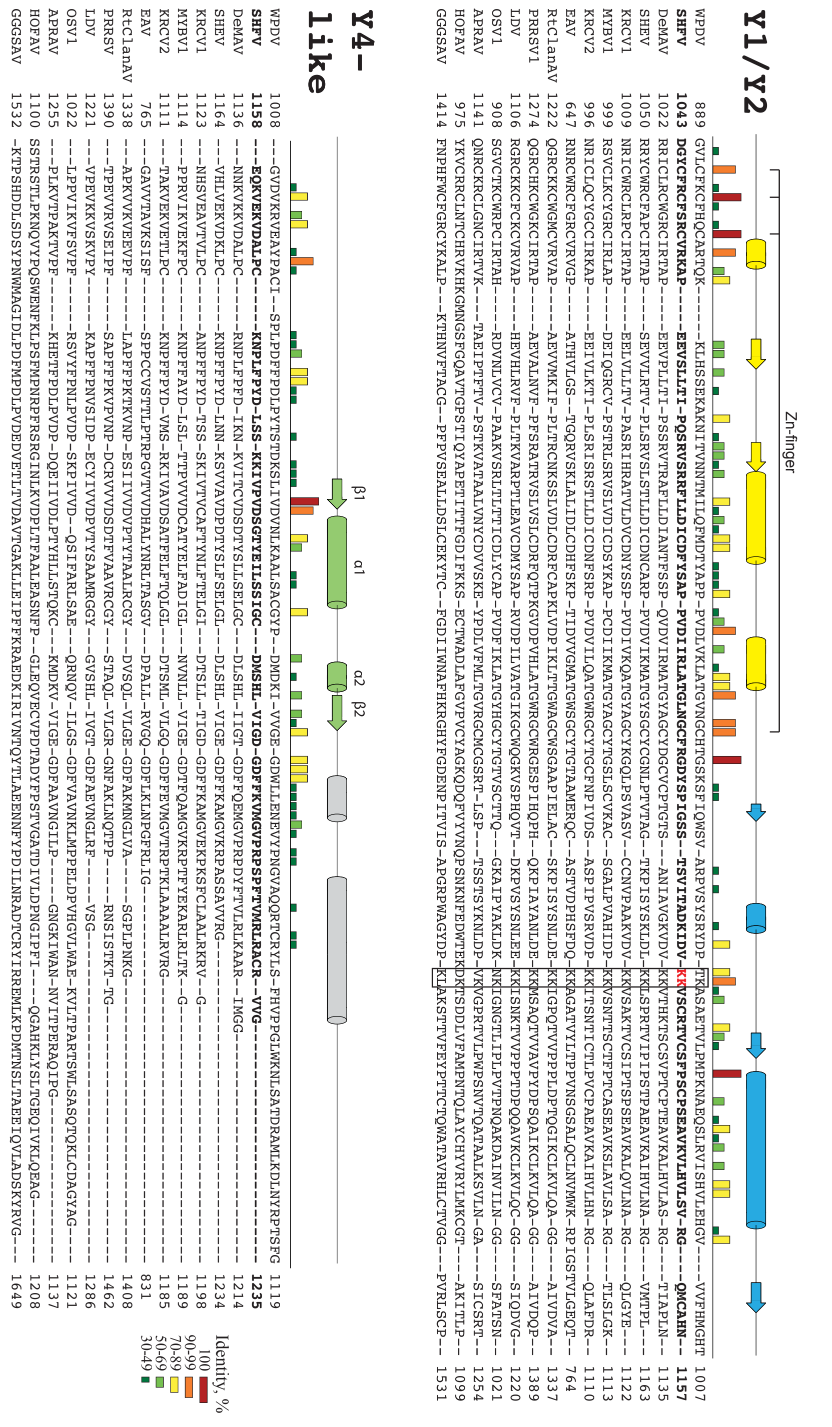

Figure S8. Sequence alignment of nsp2 Y-like regions from arteriviruses (see Table S1). The first and last residues are indicated for each species using pp1a numbering. Secondary structure elements for SHFV are schematically shown at the top. The conserved positively charged residues K1123 and K1124 of SHFV are highlighted in red, and their positions are indicated by boxes in all other arteriviruses. TheY1 and Y2

secondary structure elements are colored in yellow and blue, respectively. The single βααβ repeat is colored green.

Figure S9. Sequence alignment of TM2 regions from tobani and arteriviruses.

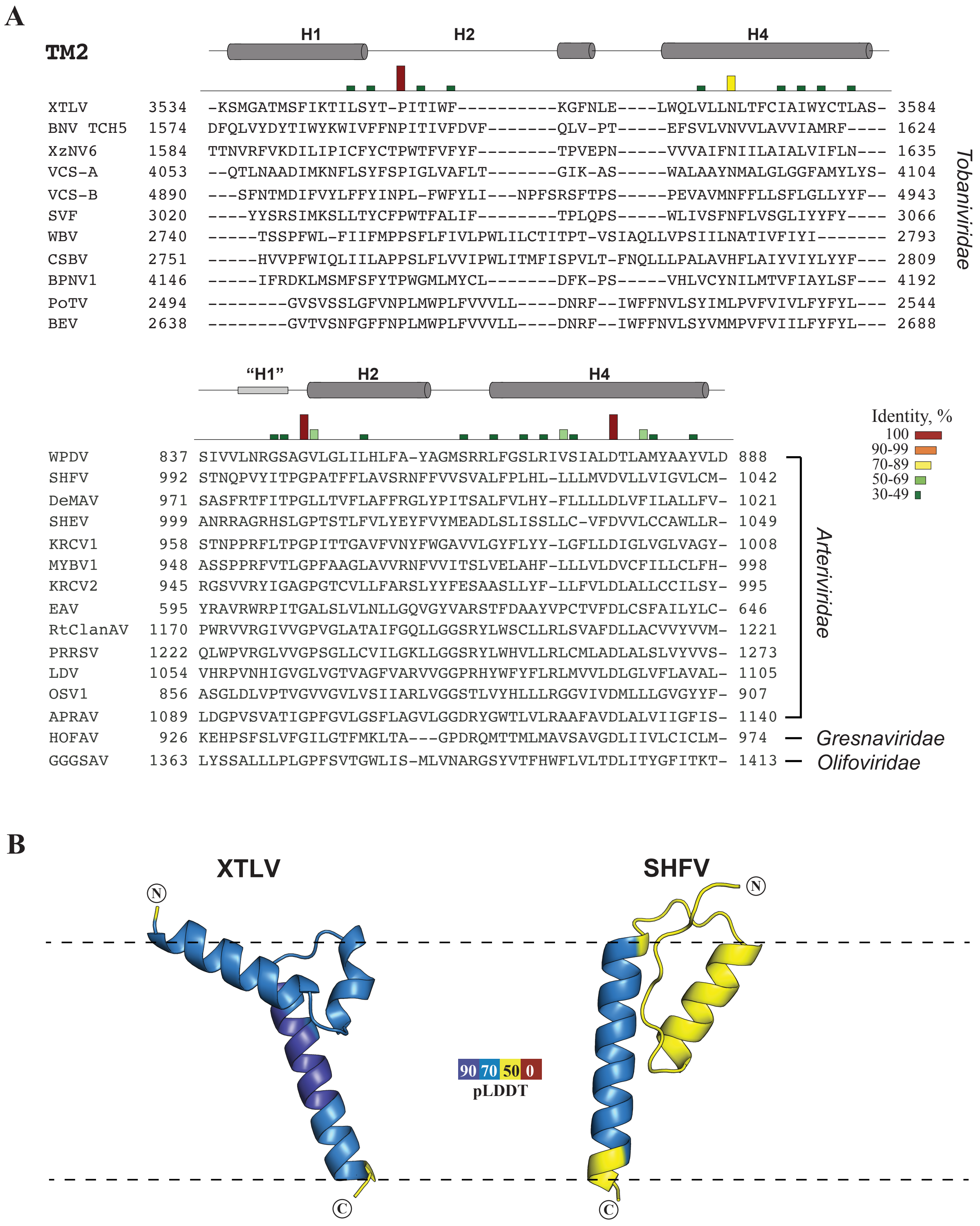

Figure S9. Sequence alignment of TM2 regions from tobani and arteriviruses.

(A) MSA of TM2 regions from all known tobani and arteriviruses (see Table S1). The first and last residues corresponding to TM2 region is indicated for each species using pp1a numbering. Helical segments of XTLV and SHFV TM2 regions are shown above the MSAs. "H1" marks the unstructured region in SHFV that is embedded in the membrane and plays role of H1 helix in corona- and tobaniviruses. The identity scores were calculated across all tested species after MSA was manually adjusted using AlphaFold structural models.

(B) Cartoon representations of AlphaFold 2 (1) models of XTLV and SHFV TM2 colored according to pLDDT scores. Dashed lines indicate the membrane boundaries predicted by the PPM 3.0 server (2).

References

1. Jumper J, Evans R, Pritzel A, Green T, Figurnov M, Ronneberger O, Tunyasuvunakool K, Bates R, Zidek A, Potapenko A, Bridgland A, Meyer C, Kohl SAA, Ballard AJ, Cowie A, Romera-Paredes B, Nikolov S, Jain R, Adler J, Back T, Petersen S, Reiman D, Clancy E, Zielinski M, Steinegger M, Pacholska M, Berghammer T, Bodenstein S, Silver D, Vinyals O, Senior AW, Kavukcuoglu K, Kohli P, Hassabis D. 2021. Highly accurate protein structure prediction with AlphaFold. Nature 596:583-589.
2. Lomize AL, Todd SC, Pogozheva ID. 2022. Spatial arrangement of proteins in planar and curved membranes by PPM 3.0. Protein Sci 31:209-220.
3. Baker NA, Sept D, Joseph S, Holst MJ, McCammon JA. 2001. Electrostatics of nanosystems: application to microtubules and the ribosome. Proc Natl Acad Sci U S A 98:10037-41.
